# Solid cancer-directed CAR T cell therapy that attacks both tumor and immunosuppressive cells via targeting PD-L1

**DOI:** 10.1016/j.omton.2024.200891

**Published:** 2024-10-05

**Authors:** Yan Luo, Martha E. Gadd, Yaqing Qie, Andrea Otamendi-Lopez, Jesus E. Sanchez-Garavito, Mieu M. Brooks, Maria J. Ulloa Navas, Tanya Hundal, Shuhua Li, Vanessa K. Jones, Yanyan Lou, Tushar Patel, Roxana Dronca, Mohamed A. Kharfan-Dabaja, Haidong Dong, Alfredo Quinones-Hinojosa, Hong Qin

**Affiliations:** 1 Regenerative Immunotherapy and CAR-T Translational Research Program, Mayo Clinic, Jacksonville, FL, USA; 2 The Neurosurgery Department, Mayo Clinic, Jacksonville, FL, USA; 3 Division of Hematology and Medical Oncology, Department of Internal Medicine, Mayo Clinic, Jacksonville, FL, USA; 4 Blood and Marrow Transplantation and Cellular Therapy Program, Mayo Clinic, Jacksonville, FL, USA; 5 Hepatology & Liver Transplantation, Mayo Clinic, Jacksonville, FL, USA; 6 Department of Urology, Mayo Clinic, Rochester, MN, USA; 7 Department of Immunology, Mayo Clinic, Rochester, MN, USA; 8 Department of Cancer Biology, Mayo Clinic, Jacksonville, FL, USA

**Keywords:** MT: Regular Issue, chimeric antigen receptor, CAR T cells, PD-L1, T cells, tumor microenvironment, solid tumor, immunotherapy

## Abstract

Chimeric antigen receptor (CAR) T cell therapy has encountered limited success in solid tumors. The lack of dependable antigens and the immunosuppressive tumor microenvironment (TME) are major challenges. Within the TME, tumor cells along with immunosuppressive cells employ an immune-evasion mechanism that upregulates programmed death ligand 1 (PD-L1) to deactivate effector T cells; this makes PD-L1 a reliable, universal target for solid tumors. We developed a novel PD-L1 CAR (MC9999) using our humanized anti-PD-L1 monoclonal antibody, designed to simultaneously target tumor and immunosuppressive cells. The antigen-specific antitumor effects of MC9999 CAR T cells were observed consistently across four solid tumor models: breast cancer, lung cancer, melanoma, and glioblastoma multiforme (GBM). Notably, intravenous administration of MC9999 CAR T cells eradicated intracranially established LN229 GBM tumors, suggesting penetration of the blood-brain barrier. The proof-of-concept data demonstrate the cytolytic effect of MC9999 CAR T cells against immunosuppressive cells, including microglia HMC3 cells and M2 macrophages. Furthermore, MC9999 CAR T cells elicited cytotoxicity against primary tumor-associated macrophages within GBM tumors. The concept of targeting both tumor and immunosuppressive cells with MC9999 was further validated using CAR T cells derived from cancer patients. These findings establish MC9999 as a foundation for the development of effective CAR T cell therapies against solid tumors.

## Graphical abstract

**Figure:**
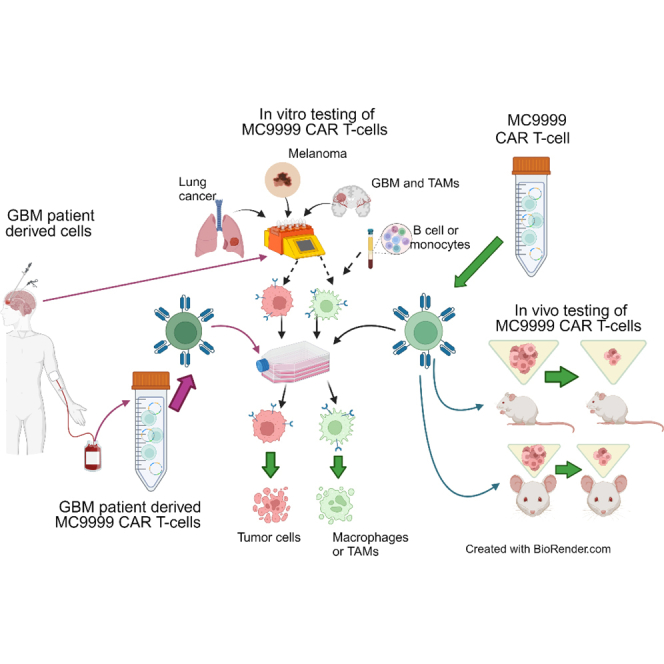

## Teaser

Novel PD-L1 targeting CAR T cells are generated and shown to recognize antigens on four solid tumor cell lines and three types of macrophage models, thus mimicking the immunosuppressive tumor microenvironment. Directly targeting cells that maintain the tumor microenvironment may be disruptive and facilitate immune cell entry into solid tumors.

## Introduction

Immunotherapies are cancer treatments that harness the immune systems of patients to fight their diseases. Two well-known examples are immune checkpoint inhibitors (ICIs) and chimeric antigen receptor (CAR) T cells. ICIs target inhibitory or stimulatory pathways that modulate immune cell activity, with many ICIs specifically mobilizing cytotoxic T cells.^1^^,^^2^ CAR T cells are a personalized therapy in which patient T cells are engineered to express a membrane spanning receptor with an extracellular domain that recognizes a cancer antigen and an intracellular domain that activates the T cell and results in an antigen-specific cellular therapy.^3^^,^^4^

CAR T cells have been revolutionary in the treatment of relapsed/refractory (r/r) B cell malignancies due to the antigen specificity to the target tissue, namely CD19, as well as the medical management of B cell aplasia.^5^ However, identification of such a target antigen is a challenge for solid tumors, particularly when considering that the presence of antigen heterogeneity complicates finding a universal antigen for CAR T cell therapy. In addition, solid tumor cells have developed complex interactions with surrounding stromal cells while recruiting and enlisting diverse immune cells to create an immunosuppressive tumor microenvironment (TME), allowing the tumor cells to escape antitumor immunity.^6^^,^^7^

A potential target that can address the hurdles of antigen heterogeneity and immunosuppression is programmed death-ligand 1 (PD-L1). The programmed cell death protein 1 (PD-1)/PD-L1 axis functions natively as a regulator of immune tolerance and as a governor for T cell activation. Cancer has manipulated this inhibitory cascade to evade T cell attack. Upregulation of PD-L1 expression on various solid tumors has been well documented,^2^^,^^8^ leading to US Food and Drug Administration approval of ICIs targeting PD-L1 for a diverse collection of solid tumors. This same T cell inhibitory mechanism can also be employed by those immunosuppressive cells within the TME, which is supported by the findings of elevated levels of PD-L1 on tumor-associated macrophages (TAMs), myeloid-derived suppressor cells (MDSCs), and regulatory T cells (Tregs).^9^^,^^10^^,^^11^ These findings highlight PD-L1 as a promising target for development of a CAR T cell therapy against both tumor cells and PD-L1-expressing immunosuppressive cells.

In this study, we developed a PD-L1 CAR T cell therapy, designated MC9999, using a humanized anti-human PD-L1 monoclonal antibody. We demonstrated the antitumor effects of MC9999 CAR T cells against various solid tumor models, including patient-derived primary tumor cell lines. Intravenously dosed MC9999 CAR T cells exhibited robust *in vivo* antitumor efficacy and long-term survival in xenograft mouse models of intramammary triple-negative human breast cancer and intracranial glioblastoma multiforme (GBM). We also showed antigen-specific cytotoxicity of MC9999 CAR T cells against three immunosuppressive cell models, including primary TAMs. To underscore the translational application of MC9999 CAR T cells, our work culminated in the successful generation of GBM patient-derived MC9999 CAR T cells that showed cytotoxicity against primary GBM tumor cells and tumor-associated microglial cells. This composite of work establishes PD-L1-targeting MC9999 CAR T cells as a promising immunotherapy with therapeutic application in solid tumors.

## Results

### Antigen-specific cytotoxicity of anti-PD-L1 CAR T cells

We used the variable regions of the heavy and light chains of a humanized PD-L1 monoclonal antibody generated by Dong et al.,^12^ which recognizes human PD-L1 and not mouse PD-L1 (Figure S1A), to create the novel MC9999 CAR. MC9999 is a second-generation CAR construct that contains the 4-1BB co-stimulatory domain and the CD3ζ signaling domain. Additionally, we included a truncated epidermal growth factor receptor (tEGFR; Figure S1B) as a safety switch that upon dosing with cetuximab would result in the elimination of tEGFR-expressing cells.^13^ MC9999 CAR expression was also confirmed with L-protein staining (Figure S1C). Our validated manufacturing procedures ensured the quality of CAR T cell production, resulting in reproducible batches of MC9999 CAR T cells that met specified quality control specifications (Table S1).^14^

**Figure 1:**
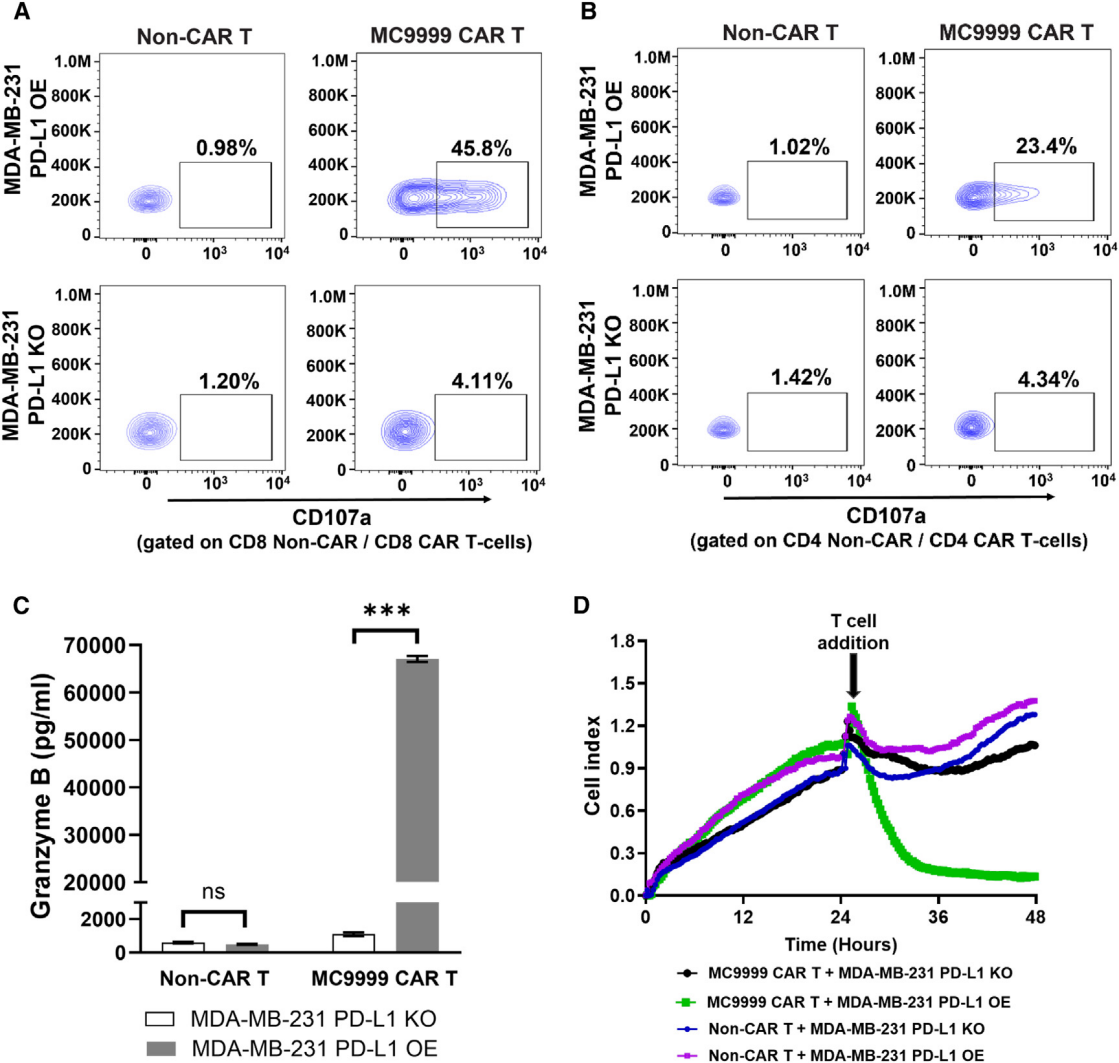
MC9999 CAR T cells showed antigen-specific cytotoxicity against PD-L1-expressing breast cancer cells (A and B) CAR T cell cytotoxicity was evaluated by CD107a surface expression in a degranulation assay. MC9999 CAR T cells were incubated with MDA-MB-231 PD-L1 OE or MDA-MB-231 PD-L1 KO cells at an E:T ratio of 2:1. Analysis was gated on CD4^+^CAR T cells (A) or CD8^+^CAR T cells (B); non-CAR T cells from the same donor served as negative controls. (C) MC9999 CAR T cells were co-cultured with either MDA-MB-231 PD-L1 OE or MDA-MB-231 PD-L1 KO cells. After 72 h, the supernatants were collected and examined for granzyme B by ELISA. The data were plotted as the mean ± SEM of quadruplicate sampling and are representative of three independent experiments (∗∗∗*p* < 0.001; ns, no significance). (D) Target cells, either MDA-MB-231 PD-L1 OE or MDA-MB-231 PD-L1 KO cells, were seeded on the electronic microtiter plates (E-plates) for 24 h. After 24 h, MC9999 CAR T cells or non-CAR T cells were added to the target cells at an E:T ratio of 40:1. CI traces were collected in triplicate every 15 min during the co-culture, and changes in impedance were normalized to the 24-h time point. The results represent three independent experiments, with a representative dataset shown.

Antigen-specific cytotoxicity of MC9999 CAR T cells was confirmed against a PD-L1 overexpressing human breast cancer cell line (MDA-MB-231 PD-L1 OE [overexpressing]) with a PD-L1 knockout variant (MDA-MB-231 PD-L1 KO) as a negative control. PD-L1 expression was confirmed on the engineered cell lines (Figure S2). Non-transduced T cells (non-CAR T cells) from the same donor were used as an alloreactivity control. Both CD8 and CD4 MC9999 CAR T cells exhibited cytotoxicity in response to MDA-MB-231 PD-L1 OE, as evidenced by T cell degranulation with subsequent surface expression of CD107a. The absence of cytotoxic activity of the same CAR T cell populations to MDA-MB-231 PD-L1 KO affirmed the antigen-specific functionality (Figures 1A and 1B, respectively). Consistently, a significant release of granzyme B was observed when MC9999 CAR T cells were incubated with MDA-MB-231 PD-L1 OE but not with MDA-MB-231 PD-L1 KO (Figure 1C). Antigen-specific cytolysis of MC9999 CAR T cells was further evaluated from the perspective of the target cells in an impedance-based killing assay. Cytolysis, as determined with the decrease in the cellular electrical impedance of cultured target cells, was observed only in MDA-MB-231 PD-L1 OE cells (Figure 1D); all other conditions showed that target cells remained intact.

**Figure 2:**
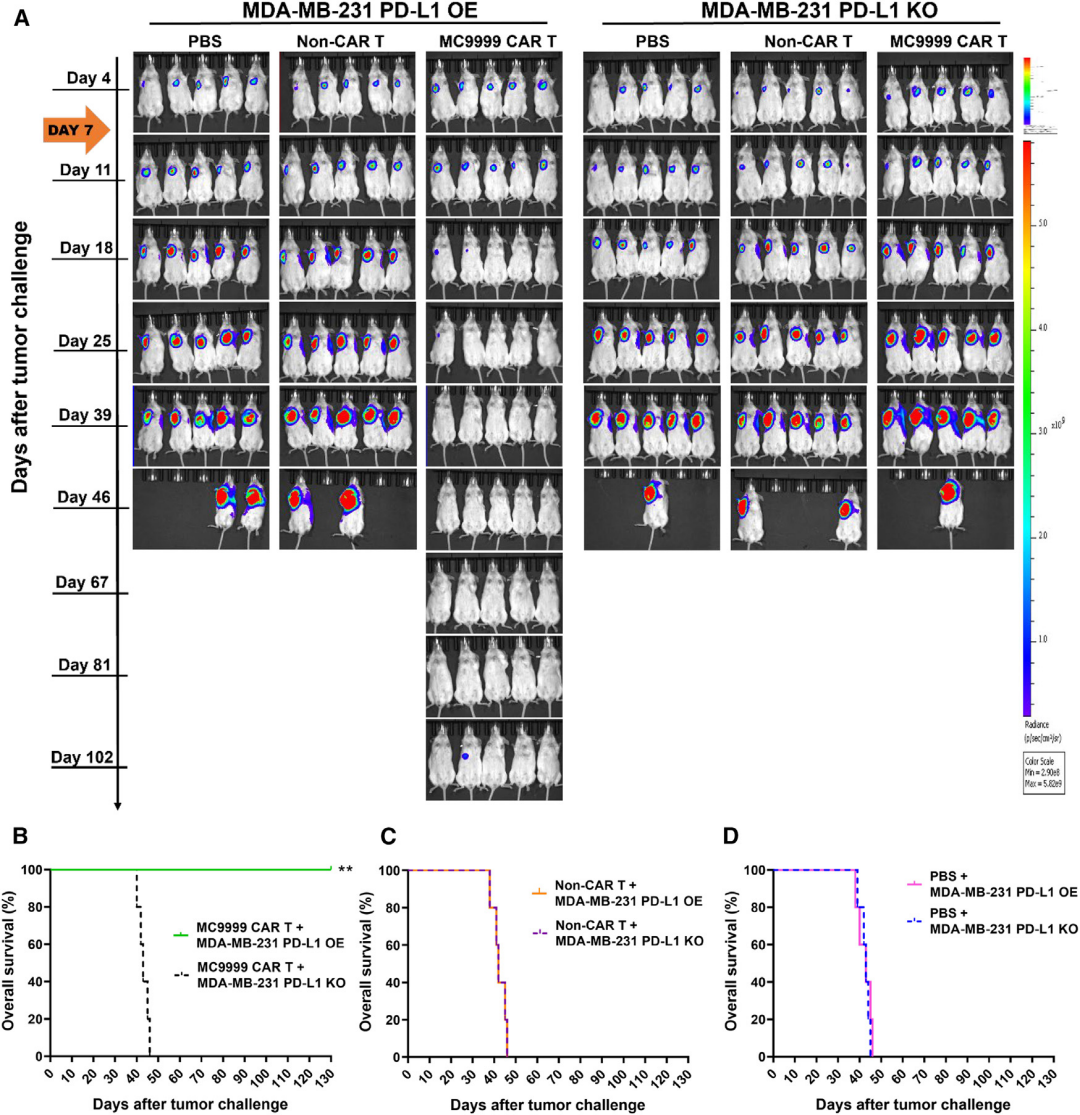
MC9999 CAR T cells elicited antitumor effects in an intramammary breast cancer model (A) Female NSG mice were given an intramammary injection of luciferase-labeled MDA-MB-231 tumor cells with either PD-L1 OE or PD-L1 KO at a dose of 1.0 × 10^6^ cells per mouse. Seven days after tumor challenge, mice were randomly divided into three groups. Each group (*n* = 5) received a single IV infusion of one of the following: PBS, non-CAR T cells (5 × 10^6^ total T cells per mouse), or MC9999 CAR T cells (2 × 10^6^ CAR T cells out of 5 × 10^6^ total T cells from the same donor/per mouse). Weekly IVIS imaging was performed to monitor tumor progression. The representative images demonstrated the changes in tumor burden over time. The results present two independent experiments using different donor T cells for generating CAR T cells. (B–D) Three separate Kaplan-Meier survival plots—MC9999 CAR T cells (B), non-CAR T cells (C), or PBS (D)—were generated to evaluate the treatment-associated overall survival. Log rank analysis revealed that MC9999 CAR T cell treatment significantly extended the overall survival in mice challenged with MDA-MB-231 PD-L1 OE comparing to those bearing MDA-MB-231 PD-L1 KO tumors (∗∗*p* = 0.01). No significant differences in survival were observed in the PBS or non-CAR T cells treatment group between these two tumor models.

We next evaluated the *in vivo* antitumor effects of MC9999 CAR T cells in female NOD scid gamma (NSG) mice that received an intramammary challenge of either MDA-MB-231 PD-L1 OE or MDA-MB-231 PD-L1 KO tumor cells, both of which were modified to express luciferase. Mice bearing established tumors were assigned to one of three treatment groups (*n* = 5 per group): MC9999 CAR T cells, non-CAR T cells, or PBS. Using bioluminescence imaging to track tumor progression, we observed a substantial reduction in established MDA-MB-231 PD-L1 OE tumors following treatment with MC9999 CAR T cells. However, this treatment showed no effect on the growth of MDA-MB-231 PD-L1 KO tumors (Figure 2A), confirming the antigen-specific *in vivo* antitumor effects. Three Kaplan-Meier plots were generated to compare treatment-associated survival rates between the antigen-positive (MDA-MB-231 PD-L1 OE) and the antigen-deficient (MDA-MB-231 PD-L1 KO) tumor challenge groups (treatment groups: MC9999 CAR T cells [Figure 2B], non-CAR T cells [Figure 2C], and PBS [Figure 2D]). Consistent with the bioluminescence imaging findings, MC9999 CAR T cell treatment resulted in prolonged survival exceeding 120 days in mice challenged with MDA-MB-231 PD-L1 OE tumor cells, while showing no such antitumor effect in those with MDA-MB-231 PD-L1 KO tumors. Neither non-CAR T cell treatment nor PBS demonstrated any antitumor effects, resulting in rapid tumor progression and mortality in all mice within 50 days post-tumor challenge.

### MC9999 CAR T cells showed antigen-specific cytotoxicity against various solid tumors

**Figure 3:**
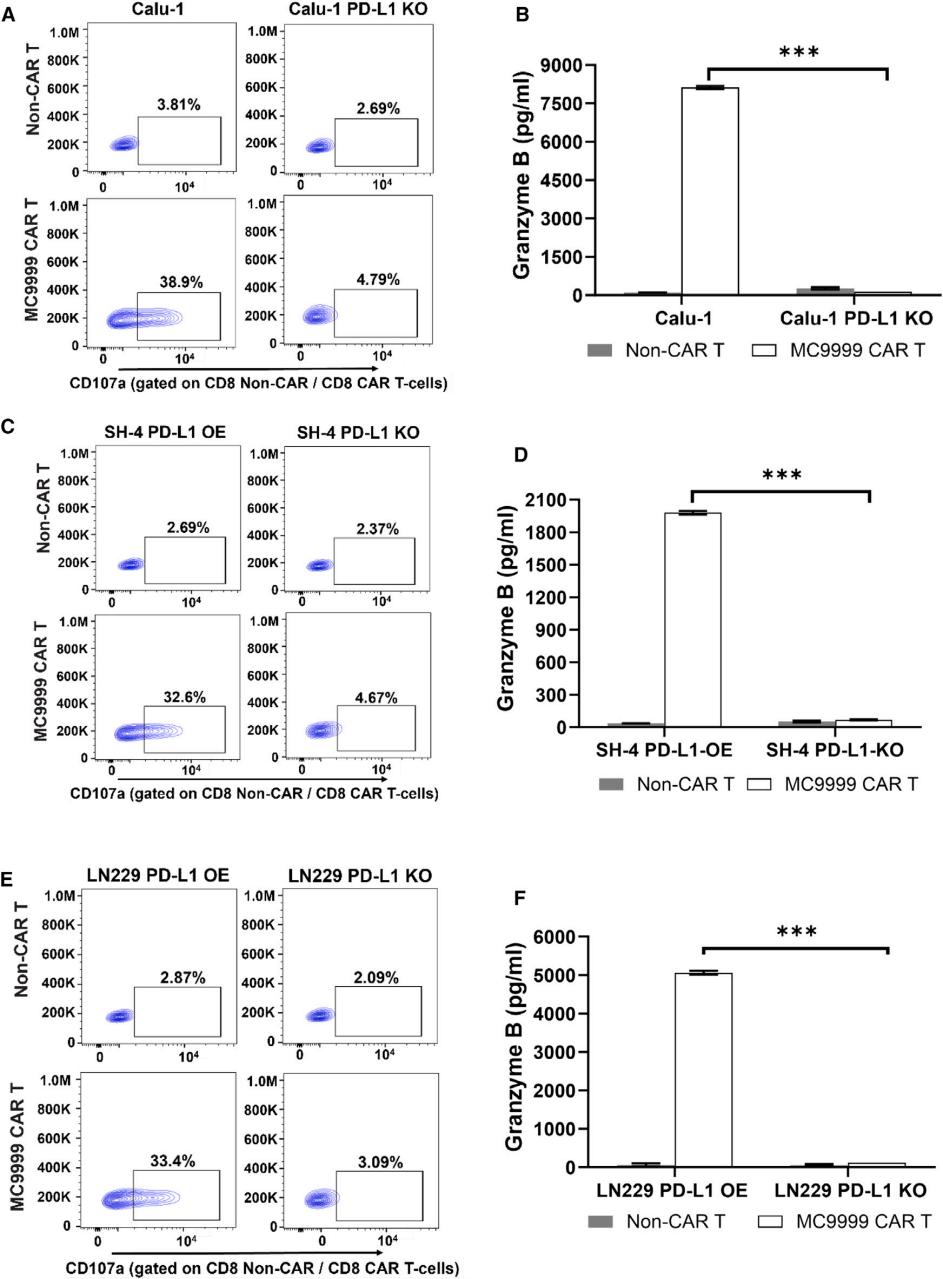
MC9999 CAR T cells exhibited antigen-specific cytotoxicity against various PD-L1 expressing solid tumors In a CD107a degranulation assay, antigen-specific cytotoxicity of MC9999 CAR T cells was assessed against three solid tumor cell lines with CD8 MC9999 CAR T cell results shown: Calu-1 lung cancer (A), SH-4 melanoma (C), and LN229 GBM (E). CD4 MC9999 CAR T cell results are included in Figure S4. The corresponding PD-L1-deficient tumor cell variants were included as negative controls. The release of granzyme B was readily detected when MC9999 CAR T cells were exposed to PD-L1-expressing target cells, including Calu-1 (B), SH-4 (D), and LN229 (F). The data, plotted as mean ± SEM of triplicate sampling, are representative of three independent experiments (*n* = 3) and analyzed using the multiple t test (∗∗∗*p* < 0.001).

Targeting PD-L1 is supported by reports of PD-L1 expression in a variety of solid tumors such as lung cancer, melanoma, and GBM. As such, we evaluated the cytotoxicity of MC9999 CAR T cells across these three types of solid tumors. Calu-1, a non-small-cell lung cancer (NSCLC) cell line, stably expresses PD-L1. We generated a PD-L1-deficient variant (Calu-1 PD-L1 KO) (Figure S3) as a control. In a CD107a T cell degranulation assay, we observed antigen-specific cytotoxicity of MC9999 CAR T cells against PD-L1-expressing Calu-1 cells (CD8 data in Figure 3A and CD4 data in Figure S4A). The release of granzyme B by the CAR T cells was exclusively detected in response to Calu-1, but not Calu-1 PD-L1 KO cells, further confirming the antigen-specific cytotoxicity (Figure 3B).

SH-4, a representative metastatic melanoma model, was engineered to generate SH-4 PD-L1 OE and SH-4 PD-L1 KO (PD-L1-deficient) cell lines (Figure S3) for use as target cells to investigate the therapeutic effectiveness of MC9999 CAR T cells against melanoma. Antigen-specific T cell degranulation was evident when MC9999 CAR T cells were incubated with SH-4 PD-L1 OE, but not with SH-4 PD-L1 KO cells (CD8 data in Figure 3C and CD4 data in Figure S4B). As further confirmation, MC9999 CAR T cells exhibited a significant release of granzyme B upon interaction with SH-4 PD-L1 OE cells (Figure 3D).

The last tumor model we examined was LN229, a GBM cell line, which was also modified to create antigen-positive LN229 PD-L1 OE and antigen-negative LN229 PD-L1 KO variants. (Figure S3). Our *in vitro* assays consistently demonstrated specific cytotoxicity of MC9999 CAR T cells against LN229 PD-L1 OE cells, evidenced by T cell degranulation (CD8 data in Figure 3E and CD4 data in Figure S4C) and granzyme B release (Figure 3F), whereas T cell cytotoxicity was absent against LN229 PD-L1 KO cells. Collectively, these findings provide conclusive evidence for the antigen-specific cytotoxicity of MC9999 CAR T cells against PD-L1^+^ solid tumors.

### MC9999 CAR T cells exhibited cytotoxicity against patient-derived GBM tumor cells

**Figure 4:**
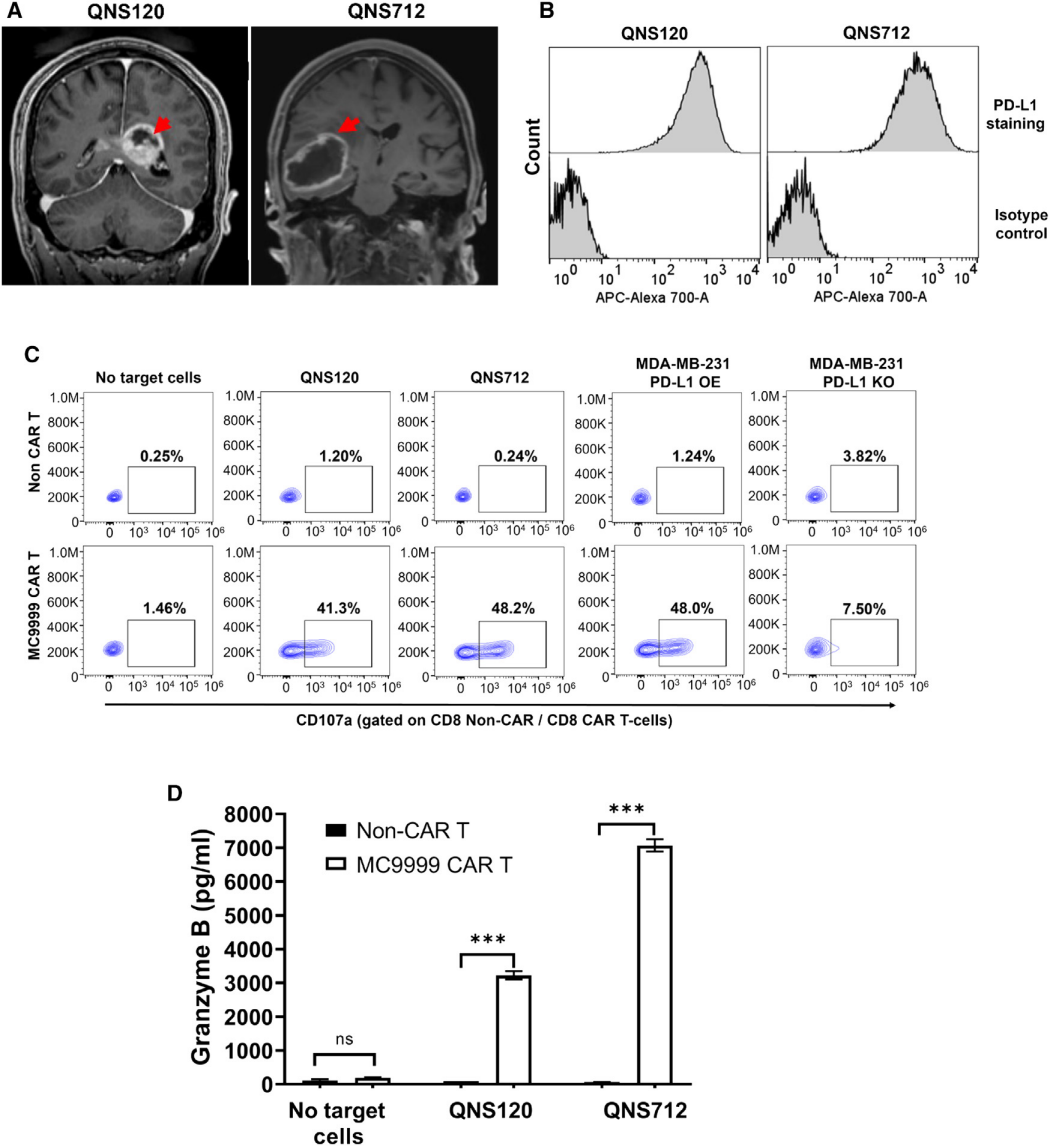
Patient-derived primary GBM cells were targeted by MC9999 CAR T cells (A) Two patients (QNS120 and QNS712) were diagnosed with glioblastoma (grade 4) as confirmed by MRI. The red arrow highlights the tumor tissues. (B) The resected tumors were obtained from the patients to generate two patient-derived primary GBM cell lines. The PD-L1 expression on QNS120 and QNS712 tumor cells was characterized using immunostaining and analyzed with flow cytometry. (C) MC9999 CAR T cells were functionally activated by QNS120 and QNS712 tumor cells, as indicated by cell surface staining of CD107a in a degranulation assay. CD8 data are shown here with the CD4 data included in Figure S5. MDA-MB-231 PD-L1 OE and MDA-MB-231 PD-L1 KO cells were used as antigen-positive and antigen-negative controls, respectively. (D) The release of granzyme B was significantly elevated when MC9999 CAR T cells targeted QNS120 and QNS712 tumor cells. The data, plotted as mean ± SEM of triplicate sampling, are representative of three independent experiments (*n* = 3) and analyzed using the multiple t test (∗∗∗*p* < 0.001).

Traditional cell lines have played crucial roles in laying the foundation for proof-of-principle CAR T cell development; however, these cell lines are devoid of the characteristics associated with their original microenvironment. Patient-derived tumor cell lines retain the tumor characteristics of the original patient and, likely the clinical response to treatment, making these primary tumor cell lines critical in translational medicine. We have established two GBM patient-derived tumor cell lines, QNS120 and QNS712, from surgically resected GBM tumors (shown in MRI images in Figure 4A), both of which are positive for PD-L1 expression (Figure 4B). MC9999 CAR T cells exhibited cytotoxicity against QNS120 and QNS712 tumor cells, as observed through CD8 T cell degranulation (Figure 4C), CD4 T cell degranulation (Figure S5), and granzyme B (Figure 4D). To ensure the elicited CAR T cell cytotoxicity was antigen specific, we included PD-L1^+^ (MDA-MB-231 PD-L1 OE) and PD-L1^−^ (MDA-MB-231 PD-L1 KO) controls. The utilization of these patient-derived GBM cell lines confirmed the therapeutic potential of MC9999 CAR T cells against primary tumor cells.

### MC9999 CAR T cell treatment eradicated intracranially engrafted GBM tumors

**Figure 5:**
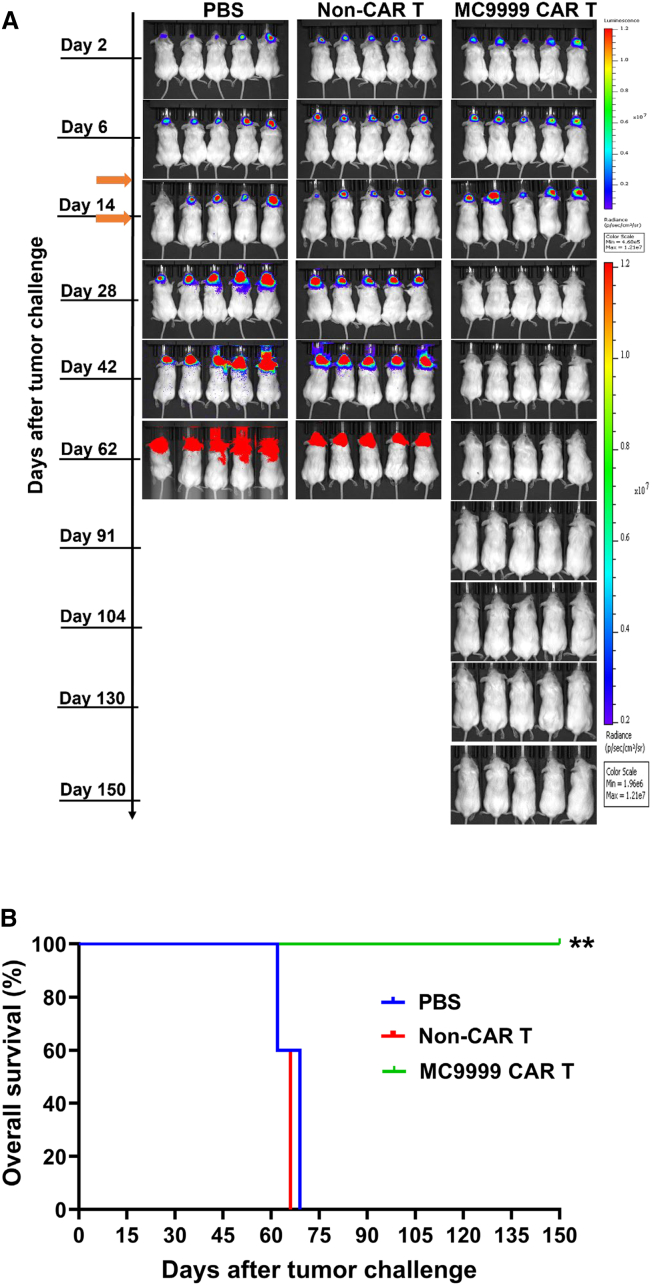
Treatment with MC9999 CAR T cells eradicated intracranially established LN229 GBM tumors (A) NSG mice were intracranially challenged with luciferase-labeled, PD-L1-OE LN229 GBM tumor cells at a dose of 0.5 × 10^6^ cells/mouse. Seven days after the tumor challenge, the mice were randomized into three groups (*n* = 5) and then received an IV infusion of one of the following: PBS, non-CAR T cells (5 × 10^6^ total T cells/mouse), or MC9999 CAR T cells (2 × 10^6^ CAR T cells out of 5 × 10^6^ total T cells/mouse) generated from the same donor. A second treatment dose was administrated IV on day 14. The mice were imaged weekly to track tumor progression for 150 days. The representative IVIS images illustrated the changes in tumor burdens over time. (B) A Kaplan-Meier plot was generated to compare the overall survival among the treatment groups. Log rank analysis revealed significant differences between the MC9999 CAR T cell treatment group and both control groups receiving either PBS or non-CAR T cells (∗∗*p* < 0.01).

We next evaluated the *in vivo* antitumor effects of MC9999 CAR T cells using the LN229 GBM tumor model (Figure 5A). Mice were challenged with an intracranial injection of luciferase-expressing LN229 PD-L1 OE cells. Treatment with either MC9999 CAR T cells, non-CAR T cells, or PBS was administered intravenously (IV) on days 7 and 14 (orange arrows) following tumor challenge. Bioluminescence images tracked tumor development and revealed significant tumor reduction in mice treated with the MC9999 CAR T cells (Figure 5A), leading to a substantially extended overall survival. The CAR T cell therapy enabled these treated mice to achieve tumor-free survival until conclusion of the experiment on day 150, whereas all mice in PBS and non-CAR T cell control groups succumbed to the tumors within 70 days after tumor challenge (Figure 5B). Our findings that IV-dosed MC9999 CAR T cells eradicated intracranially established tumors underscore the capability of these therapeutic T cells to cross the blood-brain barrier.

### Immunosuppressive cells as a target for MC9999 CAR T cells

A major challenge limiting the therapeutic efficacy of CAR T cell therapies in solid tumors is the TME, which comprises various immunosuppressive cells that highjack the PD-1/PD-L1 cascade to inhibit T cell function, allowing tumor cells to evade antitumor immunity.^2^^,^^15^ The elevated expression of PD-L1 in immunosuppressive cells makes them a potential target of MC9999 CAR T cells. We used three immunosuppressive cell models, including HMC3 microglia, monocyte-derived M2 macrophages (MDM-M2), and primary TAMs from GBM patients to test our hypothesis.

**Figure 6:**
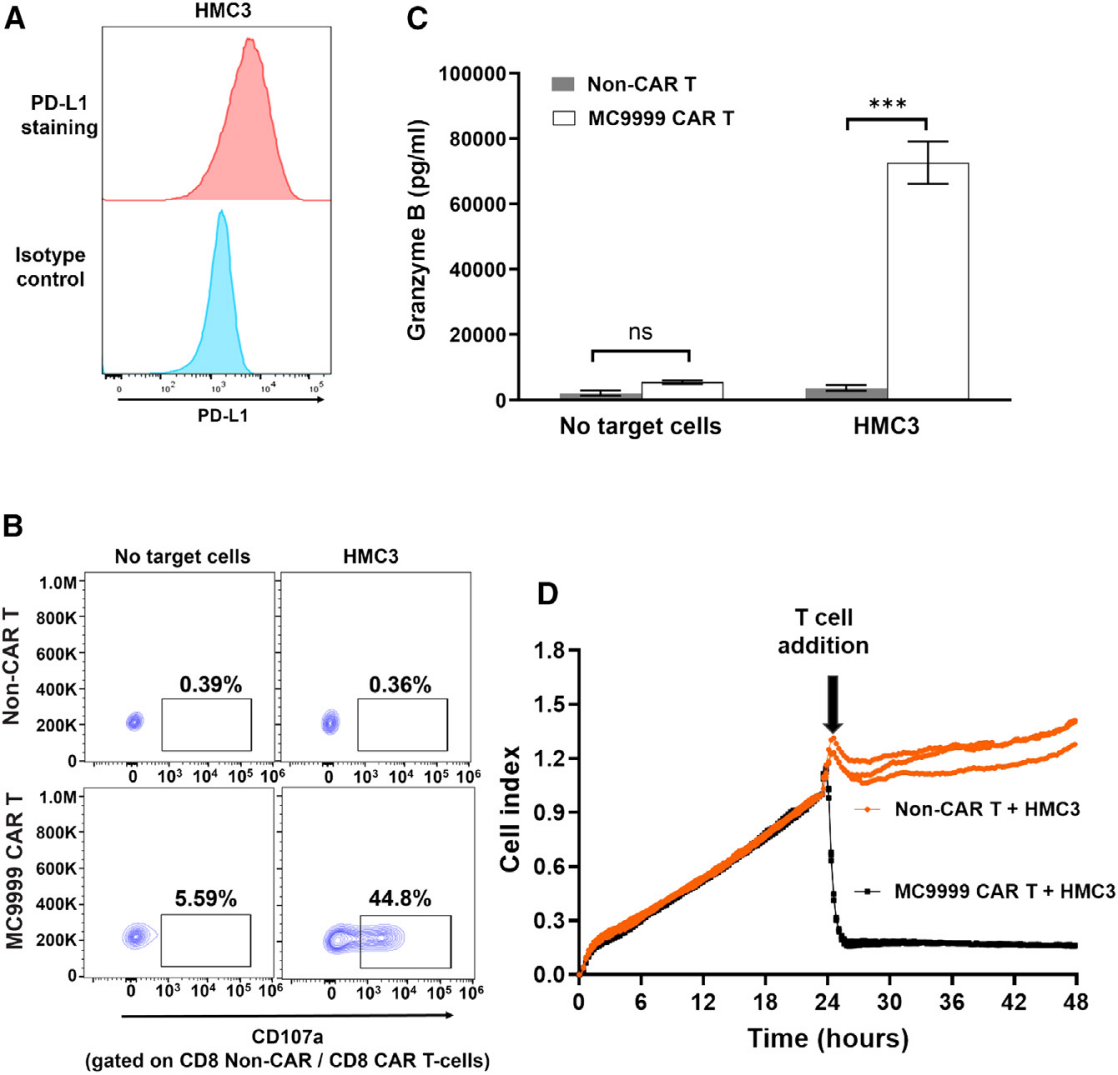
MC9999 CAR T cells elicited cytolysis of HMC3 cells modeling tumor-associated microglias (A) Immunostaining of HMC3 microglial cells with an anti-human PD-L1 monoclonal antibody demonstrated cell surface expression of PD-L1. An isotype control antibody served as a negative control. (B) Co-incubating MC9999 CAR T cells with HMC3 cells triggered T cell degranulation, as evidenced by the cell surface detection of CD107a. (CD8 data are shown here with the CD4 data included in Figure S6.) (C) A significant release of granzyme B was detected with ELISA when MC9999 CAR T cells were co-incubated with HMC3 cells. The data, plotted as mean ± SEM of triplicate sampling, are representative of three independent experiments and analyzed using the multiple t test (∗∗∗*p* < 0.001). (D) The impedance-based killing assay revealed a real-time cytotoxicity exhibited by MC9999 CAR T cells against HMC3 cells. HMC3 cells were cultured for 24 h, followed by the addition of MC9999 CAR T cells, which resulted in a significant decrease in the CI, a measure of cellular impedance, within the cultured HMC3 cells. The data are representative of three independent experiments.

Microglia residing within the TME represent a subset of TAMs in GBM.^6^ We first confirmed the expression of PD-L1 in the HMC3 microglial cell line (Figure 6A). Cytotoxicity of MC9999 CAR T cells against HMC3 cells was evident via CAR T cell degranulation (CD8 data in Figure 6B and CD4 data in Figure S6) and granzyme B release (Figure 6C). Further evidence was the direct killing of the HMC3 cells upon exposure to MC9999 CAR T cells that was measured with the disruption of the HMC3 monolayer in an impedance assay (Figure 6D).

**Figure 7:**
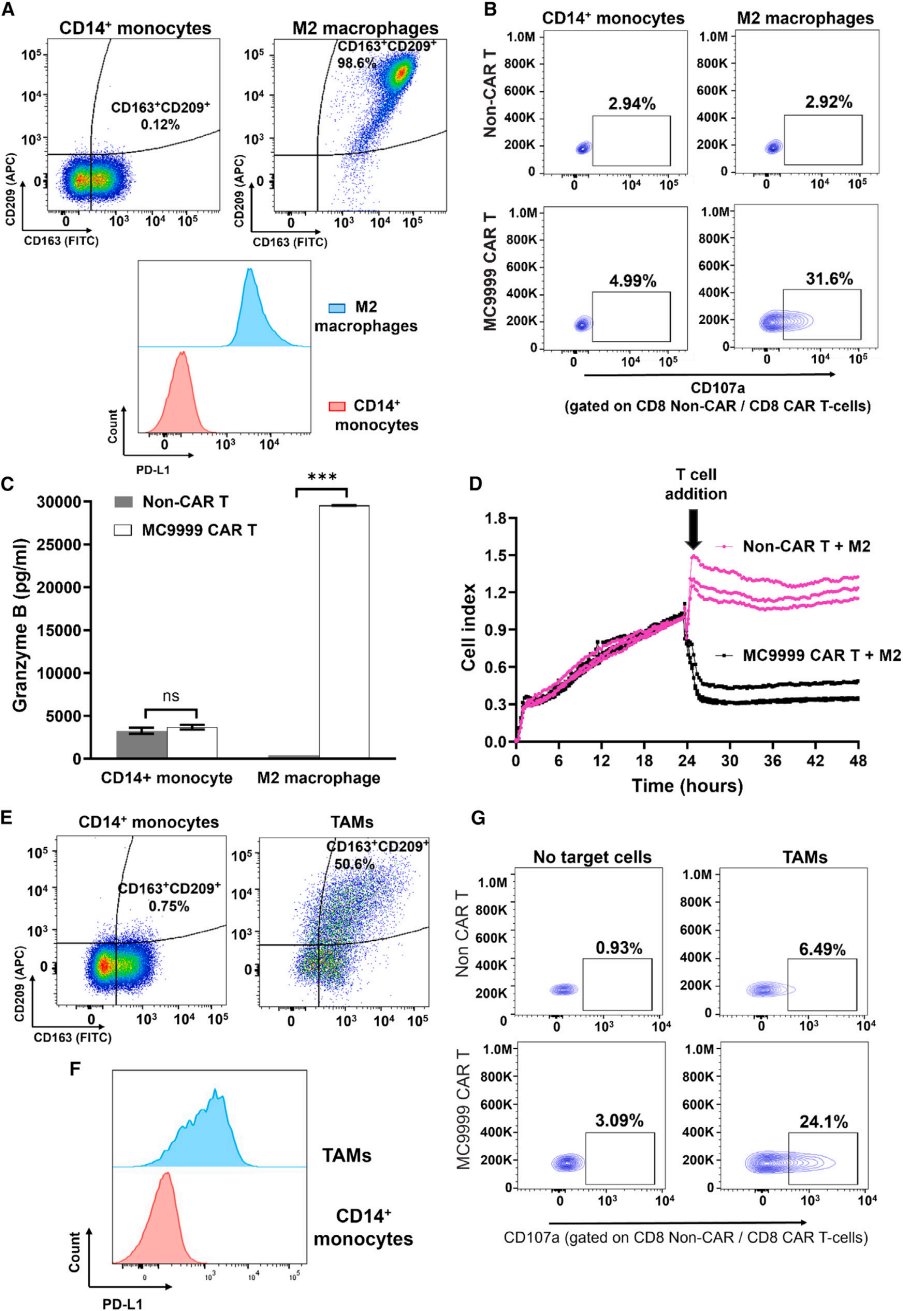
MC9999 CAR T cells target MDM-M2 that model immunosuppressive cells and patient-derived TAMs (A) The CD163^**+**^CD209^**+**^ immunophenotype of MDM-M2 was characterized and compared to that of their monocyte precursors (scatterplot). The PD-L1 expression on MDM-M2 was confirmed by immunostaining and analyzed using flow cytometry, with CD14^**+**^ monocytes serving as a negative control (histogram plots). (B) MC9999 CAR T cells exhibited cytotoxicity against MDM-M2 but not CD14^**+**^ monocytes, as determined via a CD107a degranulation assay. CD8 data are shown here with the CD4 data included in Figure S7A. (C) The release of granzyme B was significantly increased when MC9999 CAR T cells were cultured with MDM-M2. The data, plotted as mean ± SEM of triplicate sampling, are representative of three independent experiments and analyzed using the multiple t test (∗∗∗*p* < 0.001; ns, no significance). (D) The impedance-based killing assay demonstrated the direct killing of MDM-M2 by MC9999 CAR T cells, as evidenced by a significant decrease in the CI upon the addition of the CAR T cells to cultured MDM-M2. The data are representative of three independent experiments. (E) TAMs isolated from a surgically resected GBM tumor displayed the expected CD163^**+**^CD209^**+**^ immunophenotype upon immunostaining. (F) The CD163^**+**^CD209^**+**^ gated TAMs were highly positive for PD-L1 at the cell surface. CD14^**+**^ monocytes served as the negative control for both immunophenotypic characterization and PD-L1 staining. (G) Evaluation via the CD107a degranulation assay revealed that the MC9999 CAR T cells, derived from healthy donor T cells, elicited cytotoxicity against the TAMs extracted from GBM tumor. CD8 data are shown here with the CD4 data included in Figure S7B.

Immunosuppressive macrophages, including TAMs, typically exhibit an M2 phenotype within the TME. To model this population, we derived MDM-M2 and confirmed their MDM-M2 phenotype with the expression of CD163 and CD209 cell surface markers (Figure 7A, scatterplot). The expression of PD-L1 was detected on these M2 macrophages, distinguishing themselves from their CD14^+^ monocyte precursor (Figure 7A, histograms) and marking M2 macrophages as a target for MC9999 CAR T cells (CD8 data in Figure 7B and CD4 data in Figure S7A). This PD-L1 targeted cytotoxicity was further confirmed with the significant release of granzyme B by MC9999 CAR T cells upon interaction with M2 macrophages (Figure 7C). Moreover, the data from an impedance-based killing assay demonstrated the direct killing of M2 macrophages by MC9999 CAR T cells (Figure 7D).

Finally, we assessed the cytotoxicity of MC9999 CAR T cells on primary TAMs isolated from a surgically resected GBM tumor (QNS 960; Table S2). The immunophenotypic characterization verified the presence of a CD163^+^CD209^+^ double-positive TAM population from the tumor (Figure 7E). Gating on this population, we identified PD-L1 expression on these immunosuppressive cells (Figure 7F). CAR T cell degranulation was observed against TAMs (CD8 data in Figure 7G and CD4 data in Figure S7B), underscoring the potential of MC9999 CAR T cell therapy in targeting TAMs and subsequently mitigating immunosuppression within the TME of solid tumors.

### GBM patient-derived MC9999 CAR T cells targeted primary GBM tumor cells

We have shown the antigen-specific cytotoxicity of MC9999 CAR T cells against a variety of PD-L1-expressing target cells that included both cancer cells and tumor-associated immunosuppressive cells. In these proof-of-principle studies, the CAR T cells were generated from healthy donor T cells. Recognizing that T cell fitness in cancer patients may be compromised, we evaluated the cytotoxicity of patient-derived MC9999 CAR T cells (from our GBM patients) against PD-L1-expressing target cells to highlight the translational significance. Following our laboratory standard operating procedures for CAR T cell production, we generated MC9999 CAR T cells using peripheral blood T cells obtained from three GBM patients (Table S2). All three batches of patient-derived CAR T cells exhibited acceptable quality control criteria with fold expansion (expansion fold >25; Figure S8A) and the specific CAR T cell characteristics of identity (identity >70%, CD3 staining; Figure S8B) and potency (potency >10%, tEGFR staining; Figure S8C).

**Figure 8:**
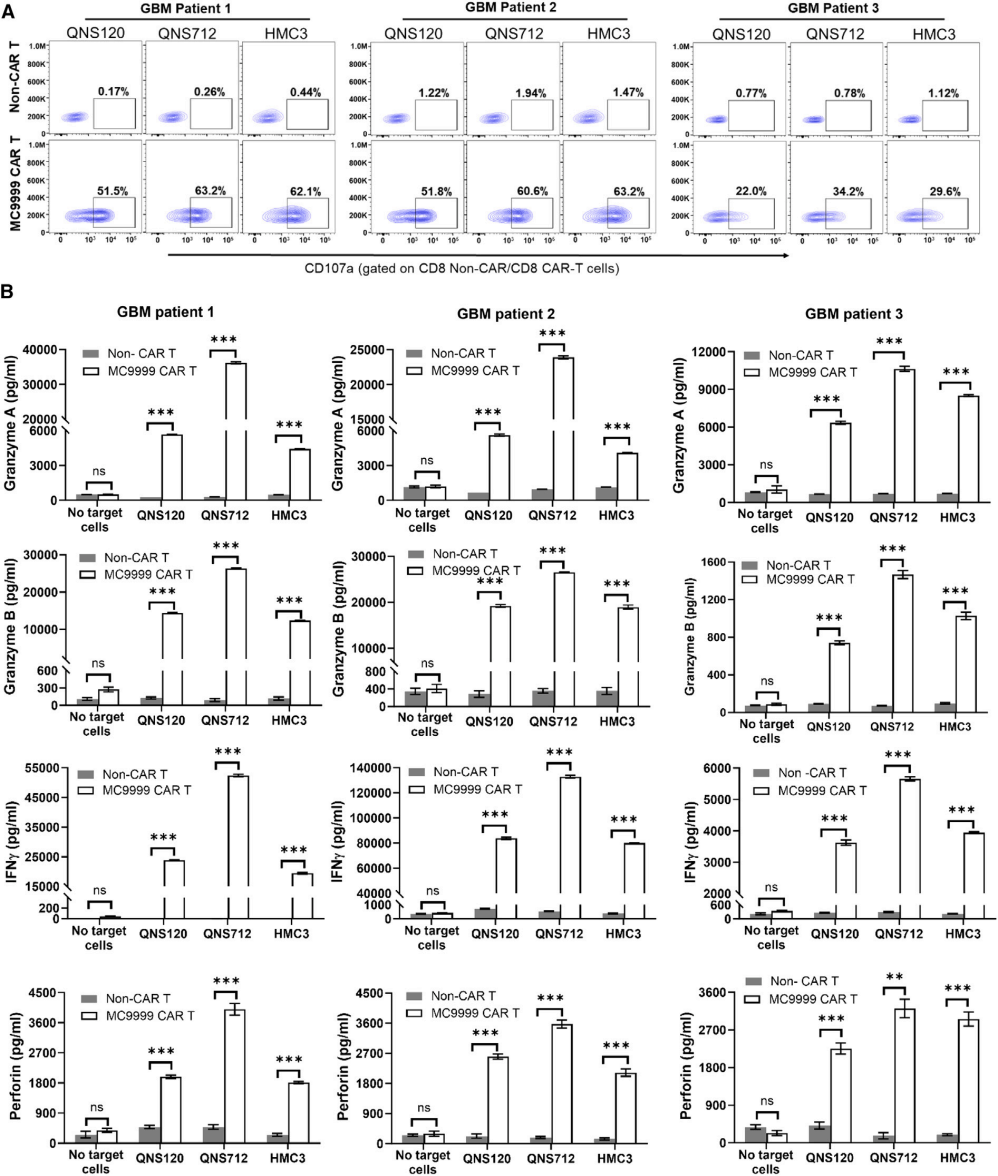
Validation of cytotoxic functionalities of GBM patient-derived MC9999 CAR T cells (A) Using peripheral blood T cells obtained from GBM patients, three batches of patient-derived MC9999 CAR T cells were generated. The cytotoxicity of these patient-derived CAR T cells was evaluated through a CD107a degranulation assay. Upon incubation with the PD-L1-expressing target cells, including QNS120 and QNS712 GBM patient-derived tumor cells, as well as HMC3 microglia cells, the CAR T cells exhibited degranulation activities, as evidenced by the presence of CD107a at the cell surface. CD8 data are shown here with the CD4 data included in Figure S9. (B) After 72 h of co-culturing patient-derived MC9999 CAR T cells with the target cells, the tissue culture supernatant was collected for quantitative analysis of T cell cytotoxic granules/cytokines, including granzyme A, granzyme B, IFN-γ, and perforin, using a customized U-PLEX Multiplex Assay. The data were plotted as mean ± SEM of triplicate sampling and analyzed using the multiple t test (∗∗*p* = 0.0011; ∗∗∗*p* < 0.001).

The antigen-specific cytotoxicity of these patient-derived MC9999 CAR T cells was evaluated against the QNS120 and QNS712 patient-derived tumor cell lines, as well as the HMC3 cell line, with the corresponding non-CAR T cells as a negative control. The CAR T cells derived from patients 1 and 2 exhibited comparable degranulation activity against the target cells, whereas those from patient 3 showed less activity (CD8 data in Figure 8A and CD4 data in Figure S9). In a separate measure of activity, we examined the release of cytotoxic granules and cytokines by the patient-derived MC9999 CAR T cells in response to target cells and detected granzyme A, granzyme B, interferon-γ (IFN-γ), and perforin in all cases (Figure 8B). As expected, variations were observed among the CAR T cells derived from different patients. CAR T cells from patients 1 and 2 released greater amounts of cytotoxic granules and IFN-γ than those from patient 3, aligning with the degranulation results. Our generation of three batches of qualified, functional patient-derived MC9999 CAR T cells shows the translational feasibility of these CAR T cells for clinical applicability.

## Discussion

To address challenges of CAR T cell effectiveness in solid tumors, we have engineered PD-L1-targeting MC9999 CAR T cells and have shown cytotoxicity and antitumor effects against various PD-L1-expressing tumor cells and macrophages that modulate the TME. The underlying rationale was that both tumor cells and immunosuppressive cells exploit PD-L1 pathways to evade immune surveillance. To this end, we employed a humanized monoclonal antibody against human PD-L1^12^ to develop a PD-L1 CAR construct, MC9999. This humanized monoclonal antibody will have a lower risk for inducing immune responses in humans compared to the original mouse monoclonal antibody; we used this novel monoclonal antibody as opposed to approved ICIs with the goal to improve responsiveness^16^ by having a novel epitope.^12^ Additionally, this humanized anti-PD-L1 antibody has a binding to PD-L1 similar to that of atezolizumab; however, the binding sites differ between these two antibodies.^12^ Using the MDA-MB-231 PD-L1 OE and MDA-MB-231 PD-L1 KO pair of cell lines, we validated antigen-specific cytotoxicity and antitumor effects of MC9999 CAR T cells in both *in vitro* and *in vivo* settings. Furthermore, MC9999 CAR T cells exhibited activity against a diverse set of PD-L1-expressing, solid tumor-derived target cells that included an NSCLC cell line (Calu-1), a melanoma cell line (SH-4), a GBM cell line (LN229), and, finally, two patient-derived GBM cell lines. The observation that IV-dosed MC9999 CAR T cells eradicated intracranially established GBM tumors is highly encouraging for the future translational application of MC9999 CAR T cell therapy for patients with GBM. Once high expression of PD-L1 was confirmed in three immunosuppressive macrophage models, we also showed the cytotoxicity of MC9999 CAR T cells against HMC3 microglial, M2 macrophages, and patient-derived TAMs. These proof-of-principle studies highlight the potential effectiveness of targeting PD-L1 with MC9999 CAR T cells against solid tumors.

Immunotherapies, specifically CAR T cells and ICIs, mobilize the patient’s immune system to battle cancer. Indeed, CAR T cell therapies have revolutionized the treatment of hematological cancers, with complete remissions reported as high as 71%–81% for r/r acute lymphoblastic leukemia.^17^ This success story inspires researchers to continue the quest for a CAR T cell therapy for the treatment of solid tumors, even though this success has yet to be fully realized. Challenges remain, specifically with solid tumors having heterogeneous antigens or lacking a restrictive antigen, and issues with trafficking into tumor and retaining activity of CAR T cells once in the immunosuppressive TME.^18^ The identification of the immune checkpoints that regulate T cell function birthed ICIs, with a prominent targeted pair of PD-1 and its ligand, PD-L1.^1^ We were particularly drawn to the PD-1/PD-L1 cascade with the well-characterized relationship between increased PD-1 expression and suppressed T cell function along with the observation that tumor cells and regulatory/support cells both can express PD-L1 to evade T cell-mediated antitumor immunity. The presence of PD-L1 is a negative prognostic marker,^19^^,^^20^ with reports of circulating PD-L1^+^ monocytes associated with some cancers.^21^^,^^22^^,^^23^ Additionally, combinations of ICIs and CAR T cell therapies are being proposed to improve CAR T cell functions.^24^^,^^25^ Hence, we hypothesized that designing PD-L1 targeting CAR T cells would not only be able to strike tumor cells but would also attack the PD-L1-expressing regulatory/support cells, resulting in the destabilization of the cellular network that generates the immunosuppressive TME.

This immunosuppressive TME has a cellular composition that forms a cellular network in which regulatory and support cells control access, modulate activation, and suppress activity of immune cells, specifically cytolytic T cells and tumor infiltrating lymphocytes, which are tasked with eradicating tumor cells.^26^ Initially, classical immunohistochemistry and more recently advanced methods like spatial transcriptomics contributed to the representative image of solid tumors,^27^^,^^28^^,^^29^ with macrophages sometimes occupying more than 50%.^30^ Elevated PD-L1 levels have been consistently observed in various immunosuppressive cells within the TME, including TAMs, MDSCs, and even Tregs, which makes PD-L1 a viable therapeutic target for these immunosuppressive cells. With this perspective in mind, the MC9999 CAR was uniquely engineered to target not only tumor cells but also immunosuppressive cells within the TME for more effective therapeutic outcomes. We tested our hypothesis by validating the cytotoxicity of MC9999 CAR T cells against three immunosuppressive macrophage models that highly express PD-L1, including HMC3 microglial cells, MDM-M2s, and patient-derived TAMs. These proof-of-principle studies highlighted the potential effectiveness of targeting PD-L1 with MC9999 CAR T cells against the TME.

The success of PD-1/PD-L1 ICIs for the treatment of malignancies is remarkable but not universal, and the generation of PD-L1 targeted CAR T cells that allows for a more permanent elimination of PD-L1-expressing target cells remains a goal. Our findings solidify MC9999 CAR T cells as a valid immunotherapy option with broad application to solid tumors with activity against not only tumor cells but also immunosuppressive cells within the TME. Generating MC9999 CAR T cells from the peripheral T cells of GBM patients is highly encouraging, particularly after measuring their activity against both microglial cells and patient-derived GBM cell lines. We are actively developing a novel approach that leverages the operating room for the intratumoral delivery of CAR T cells, which allows effective T cell application directly to tumor sites. We are encouraged by the response of MC9999 CAR T cells against diverse cell lines since GBM, NSCLC, and melanoma have antigen heterogeneity while also expressing PD-L1 to induce immunosuppression.^31^ CAR T cell therapies still face several challenges specifically related to the single-chain variable fragment (scFv) that are part of their structures. We did not observe graft-versus-host disease (GVHD) symptoms in the reported mice models across the long treatment time; however, we hypothesize that our dosages of 5 × 10^6^ total T cells were below the quantity of cells to induce GVHD in these mouse models, as we have observed this adverse event in other cohorts of animals that received human CAR T cells.

Even with these promising proof-of-concept data, we also acknowledge several remaining challenges before our goal can be actualized. Perhaps the most concerning issue related to any PD-L1-targeted CAR T cell is the safety profile, particularly the risk of on-target, off-tumor effects that may lead to adverse clinical events. We are addressing several avenues to mitigate these potential risks. First, we are using a humanized monoclonal antibody that will lower the risk for inducing immune responses in humans compared to the original mouse monoclonal antibody. Although mouse PD-L1 and human PD-L1 share low sequence identity, the interactional surface with the PD-1 ligand has a similar arrangement, allowing for interspecies binding; however, antibodies that were generated from either mouse PD-L1 or human PD-L1 should undergo *in vitro* characterization against both mouse and human targets.^32^ We confirmed the functional specificity of our humanized PD-L1 antibody against human PD-L1. Second, we are utilizing the scFv from a novel anti-PD-L1 antibody^33^ that was humanized and binds an epitope that is distinct from atezolizumab.^12^ Other PD-L1 CAR T cells generated from the existing atezolizumab monoclonal antibody^34^^,^^35^ or from novel monoclonal^36^^,^^37^ or humanized PD-L1-targeting antibodies have been characterized and showed promising preclinical results.^38^ However, a phase 1 clinical trial exploring PD-L1 CAR T cell therapy for NSCLC resulted in a serious adverse event of pulmonary toxicity that developed 47 days post-CAR T cell infusion.^39^ This observation in a clinical trial and other reports^34^^,^^40^ suggest that PD-L1 targeted CAR T cell therapies have the potential for off-target effects that require additional investigation.

To this end, we are optimizing the safety of MC9999 CAR T cell therapy with strategies that include the localized as opposed to systemic CAR T cell delivery to minimize potential side effects as well as upgrades to our CAR design. We are also exploring the feasibility of intracranial delivery of MC9999 CAR T cells during neurosurgery for the treatment of GBM. Our current MC9999 CAR construct incorporates tEGFR as a safety switch, enabling the depletion of CAR T cells upon the introduction of cetuximab.^13^ Another strategy related to CAR construct design is our modeling and engineering of low-affinity variants of our PD-L1 antibody that will spare normal tissues with low levels of antigen expression yet retain binding affinity to target tumor cells that overexpress antigen. Lastly, we are also considering a SynNotch CAR design strategy^41^^,^^42^ to couple our MC9999 CAR with a more tumor-specific CAR, aiming to enhance tumor-specific targeting of MC9999 CAR T cells. These safety-focused strategies will aid in the translation of a safe MC9999 CAR T cell therapy into clinical applications for treating solid tumors.

## Materials and methods

### Cell lines and culturing conditions

The cell lines of MDA-MB-231, Calu-1, SH-4, LN229, HMC3, 293FT, and Jurkat were purchased from American Type Culture Collection (USA) and maintained in either 90% RPMI 1640, Iscove’s Modified Dulbecco’s Medium, or 90% DMEM (Thermo Fisher, USA) supplemented with 10% heat-inactivated fetal bovine serum (Thermo Fisher). Cell lines were authenticated by flow cytometry. The PD-L1 OE variant cell lines of MDA-MB-231 PD-L1 OE, LN229 PD-L1 OE, and SH-4 PD-L1 OE were generated as previously described.^14^ PD-L1 KO was induced in using IDT’s CRISPR-Cas12a (Cpf1) format involving ribonucleoprotein complex between *Acidaminococcus* Cas12a enzyme and PD-L1 guide RNA (gRNA) (TATTCATGACCTACTGGCATT) targeting exon-2 of PD-L1 following IDT product instructions. For PD-L1 KO in MDA-MB-231, LN229, Calu-1, and SH-4, the following 4D-Nucleofector programs were chosen: CH125, DS138, EO120, and EH100, respectively. KO cell lines were established from single-cell clones post-flow sorting. Luciferase-expressing human cell lines for *in vivo* experiments were generated as described.^14^ Prior to cryopreservation, the antigen-specific cell lines were authenticated using flow cytometry. All cell lines were routinely tested for mycoplasma contamination.

### PBMCs and Tn/mem isolation from healthy donors’ blood samples

Peripheral blood mononuclear cells (PBMCs) were obtained from healthy volunteer donors via leukapheresis using leukocyte reduction system cones, by the Division of Transfusion Medicine, Mayo Clinic (Rochester, MN), following current regulatory requirements and as previously described.^43^ To generate CAR T cells, naive and memory T cell (Tn/mem) populations were isolated from PBMCs in a three-step procedure, involving negative selection of both CD14 and CD25, followed by positive selection of CD62L, using CD14, CD25, and CD62L microbeads, adhering to the manufacturer’s protocol (Miltenyi Biotech, Germany).

### Isolation of T cells from blood samples of patients with GBM

The GBM patient blood procurement was performed under the biorepository protocol (institutional review board [IRB] no. 17-003013) approved by the Mayo Clinic in Florida IRB. All patients provided written informed consent, and the protocol adhered to the ethical principles of the Declaration of Helsinki. A single approximate 20-mL blood sample was collected. Disease characteristics of these patients were recorded (Table S2). T cells were isolated using the Pan T cell isolation kit (Miltenyi Biotec).^14^

### CAR T cell generation

A second-generation PD-L1-CAR (MC9999) was designed consisting of a novel PD-L1 antibody scFv,^12^ a hinge region with a CD4 transmembrane domain, and 4-1BB and CD3ζ intracellular signaling domains, complemented with a tEGFR (Figure S1). The CAR cDNA was integrated into pHIV.7 lentiviral vector. To ensure efficient lentivirus production, we utilized 293FT cells, followed by concentration and titer determination using Jurkat cells.

Tn/mem or pan-T cell populations, isolated from PBMCs, were divided into two aliquots for generating non-CAR T cells as control and another for generating CAR T cells. The detailed CAR T cell production protocol and accompanying quality control assays follow established methods.^14^ Briefly, Tn/mem or subject T cells were isolated and activated with Human T-Activator CD3/CD28 beads (Life Technologies, USA) for 24 h, followed by protamine sulfate-enhanced transduction with lentivirus encoding CAR at an optimized MOI. The CAR T cells were further activated with CD3/CD28 bead stimulation for 6 days, after which the beads are removed, and the CAR T cells were expanded for an additional 7 days. Non-CAR T cells are nontransduced T cells from the same donor, expanded following the CAR T cell protocol, and used as a control. GBM patient-derived MC9999 CAR T cells were generated using the same method using pan T cells due to the technical challenges of isolating sufficient Tn/mem cells from limited patient blood samples.^14^

### BTIC isolation and expansion from tumor tissue

Patient tumor tissue was collected with informed consent and approved by the Mayo Clinic in Florida IRB (no. 16-008485). Brain tumor-initiating cells (BTICs) were isolated and expanded from primary patient glioblastoma tumor samples and were cultured under normoxic conditions (37°C, 5% CO_2_, 20% O_2_), following previously established methods.^44^^,^^45^^,^^46^^,^^47^^,^^48^^,^^49^^,^^50^ QNS120 and QNS712 are two such cell lines. In brief, the process involved the intraoperative resection of GBM tumor tissue, which was immediately delivered to the lab for processing. The tissue was mechanically dissociated, followed by enzymatic digestion using TrypLE-express (Thermo Fisher) to separate tumor cells from other tissue. The separated cells were then passed through a 40-μm nylon mesh, collected in a 50-mL conical tube, and centrifuged at 200 × *g* for 5 min. After aspirating the supernatant, the cell pellet was resuspended in 1 mL stem cell media consisting of DMEM/F12 (Gibco, USA), 1% v/v Anti-anti (Sigma, USA), 2% v/v Gem21 NeuroPlex Serum-Free (without vitamin A) (Gemini Bio-Products, USA), fibroblast growth factor (FGF) (20 ng/mL, PeproTech, USA), and EGF (20 ng/mL, PeproTech). After re-suspending, cell count and viability were measured using the Vi-Cell XR Cell Viability Analyzer (Beckman Coulter, USA). The cells were seeded as single-cell suspensions in non-adherent culture flasks at a density of 1.2 × 10^4^ cells/cm^2^. These cells were cultivated as oncospheres for three passages before being plated and expanded on laminin-coated flasks for the establishment of cell lines. Throughout this process, all cells were maintained in stem cell-promoting media containing EGF and FGF.

### MDM-M2 from PBMCs

CD14^+^ monocytes were isolated using positive selection with CD14 microbeads (Miltenyi Biotech). Macrophages were generated from this CD14^+^ population by activation with macrophage colony-stimulating factor (M-CSF) (25 ng/mL, PeproTech) over a 7-day period. On day 7, M2 polarization was induced by the addition of interleukin-4 (20 ng/mL, PeproTech), in conjunction with M-CSF, for an additional 48–72 h.^51^ Following M2 polarization, the cells were harvested, and their phenotype and PD-L1 expression were subsequently assessed via Fortessa flow cytometry (BD, USA) by staining with BUV395-CD14 (clone M5E2, BD Horizon, USA), APC-CD209 (clone DCN46, BD Pharmingen, USA), AF488-CD163 (clone MAC2-158, BD Pharmingen), and BV650-PDL-1 (clone29E.2A3, BioLegend, USA).

### Isolation of TAMs from tumor tissues of patients with GBM

GBM patient tumor tissue was collected with informed consent (IRB no. 16-008485). The tumor tissue was first mechanically dissociated and further processed with the Tumor Dissociation Kit (catalog no. 130-095-929, Miltenyi Biotech) and the gentleMACS Dissociators (Miltenyi Biotech) for single-cell dissociation. The separated cells were then passed through a 40-μm nylon mesh, collected in a 50-mL conical tube, and centrifuged at 300 × *g* for 7 min. After aspirating the supernatant, the cell pellet was resuspended in 10 mL PBS for lymphocyte cell isolation using a Ficoll gradient centrifuge, following previously established methods.^14^ T cells were removed by negative selection, and the remaining cells were analyzed via Fortessa flow cytometry (BD). The analysis included staining for BUV395-CD14 (clone M5E2, BD Horizon), APC-CD209 (clone DCN46, BD Pharmingen), AF488-CD163 (clone MAC2-158, BD Pharmingen), and BV650-PDL-1 (clone29E.2A3, BioLegend).

### *In vitro* functional assays

#### Degranulation assays

CAR T- cells were incubated with target cells at an effector-to-target (E:T) ratio of 2:1 in complete RPMI 1640 medium containing GolgiStop Protein Transport Inhibitor Reagent (BD) and CD107a APC antibody (BD) for 6 h, following optimization of conditions to maximize CD107a surface detection.^52^ The cells were subsequently stained with anti-CD3 BV605 (BD), anti-CD4 PE-Cy7 (BD), anti-CD8 APC-Cy7 (BD), and anti-EGFR BV421(BD). Samples were evaluated using the Attune flow cytometer (Thermo Fisher Scientific) or the Fortessa flow cytometer (BD), and data were analyzed using FlowJo version 10 software. Non-CAR T cells from the same donors were used as negative controls. Experiments were performed three times, and presented data are representative.

#### Granule release assay

Following optimization of conditions to detect maximal cytokine release, CAR T cells and target cells were co-incubated for 72 h at an E:T ratio of 4:1. After the incubation period, the supernatant was collected and evaluated for granule protein release. The levels of granule proteins involved in cytotoxic T cell activity, specifically, granzyme A, granzyme B, IFN-γ, and perforin, were quantified using a customized U-PLEX Multiplex Assay kit, following the manufacturer’s instructions (Meso Scale Diagnostics, USA). Experiments were performed in triplicate with three or four replicates per ELISA plate (specified in the figure legends); GraphPad Prism was used to perform statistical analysis and to generate figures. Error bars are included; however, several SEMs are small and difficult to resolve from the outline of the data bar.

### Impedance-based tumor cell killing assay (xCELLigence)

All experiments were performed using the respective target cell culturing media. The seeding of target cells was performed in 100 μL medium per well to E-Plates 96 (Roche, Germany), and appropriate cell densities were determined through titration experiments. Cell attachment was continuously monitored using the RTCA SP instrument (Roche) and RTCA software version 1.1 (Roche) until the plateau phase was reached, typically occurring after approximately 24 h. Subsequently, T cells were introduced at an E:T ratio of 40:1 in 100 μL medium. Impedance measurements were measured every 15 min for a duration of up to 96 h. Each experiment was conducted in triplicate and performed in three separate experiments. Changes in electrical impedance were quantified as a dimensionless cell index (CI) value, which is derived from relative impedance changes corresponding to cellular coverage of the electrode sensors, normalized to baseline impedance values with medium only. For data analysis, CI values were exported, and the percentage of lysis was calculated relative to the control cells alone.

### Animal studies with bioluminescence imaging

NSG mouse breeding pairs were purchased from The Jackson Laboratory (stock no. 005557) to establish a breeding colony that was monitored in a pathogen-free animal facility at the Animal Resource Center at Mayo Clinic in Florida, per institutional guidelines. Animal studies were approved by and in accordance with guidelines of the Institutional Animal Care and Use Committee (15020; protocol nos. A00005759 and A00006674). Mice (8–12 weeks old) received an IV^53^ with a luciferase-expressing human tumor cell line (optimized in a separate experiment), randomized into test groups (5 mice per group), and treated with a single IV treatment dose of one of three treatments: PBS, non-CAR T cells, or CAR T cells (concentrations in specific figure legends). For the GBM model, mice (8–12 weeks old) received an intracranial challenge^47^^,^^50^ with a luciferase-expressing human tumor cell line (optimized in a separate experiment), randomized into test groups (5 mice per group), and had two IV treatments spaced 1 week apart; treatment was either PBS, non-CAR T cells, or CAR T cells. The tumor burden was quantified weekly by bioluminescent signal intensity on isoflurane-anesthetized mice that received a subcutaneous injection of D-luciferin (150 μg luciferin/1 g mouse body weight) 10 min prior to *in vivo* imaging system (IVIS) imaging (PerkinElmer, USA). Survival data were presented and reported in Kaplan-Meier plots.^53^

## Data and code availability

The raw data generated and analyzed to generate the published results are available upon request from the corresponding author.

## Acknowledgments

We would like to acknowledge the funding support to H.Q., which includes the 10.13039/100006827Florida Health Grant (#MOG07, SB2500), the Mayo Clinic in Florida CAR-T Manufacturing Program Fund, the Florida Department of Medicine Team Science Award, and the Mayo Clinic President’s Discovery Translation Program Award. The authors thank the Animal Resource Center at Mayo Clinic in Florida for the daily care of mice used in this study, the flow cytometry facilities at Mayo Clinic in Florida, and the Neurosurgery Biospecimens Repository of Intraoperative Patient Donations to Foster Collaborations across the Globe and Enterprise (BRIDGE, https://www.mayo.edu/research/labs/brain-tumor-stem-cell-research/research/neurosurgery-bridge-biobank). This publication was made possible through the support of the Distinguished Mayo Clinic Investigator Award (to A.Q.-H.) and the William J. and Charles H. Mayo Professorship (to A.Q.-H.), the Uihlein and Jacoby Neuro-oncology Research Fund (to A.Q.-H.).

## Author contributions

H.Q., Y.L., and A.Q.-H. designed the project and studies. J.E.S.-G., M.M.B., M.J.U.N., A.O.-L., and V.K.J. developed GBM patient-derived tumor cell lines and conducted *in vivo* studies on GBM models. Y.L., Y.Q., T.H., and S.L. participated in the development of the MC9999 CAR construct, generating experimental models, performing CAR T cell experiments, and analyzing resulting data. H.Q., M.E.G., and Y.L. created figures and prepared the manuscript. H.D. developed the anti-human PD-L1 monoclonal antibody and contributed to the conception of the MC9999 CAR T cell therapy. Y.L., T.P., R.D., M.A.K.-D., and A.Q.-H. contributed to the disease-specific experimental design, data analysis, and manuscript review. H.Q. and A.Q.-H. supervised the entire project.

## Declaration of interests

M.A.K.-D. discloses research/grants from Bristol Myers Squibb, Novartis, and Pharmacyclics, and a consultancy for Kite Pharma.

## Supplemental information

Document S1. Figures S1–S9 and Tables S1 and S2

Document S2. Article plus supplemental information
