# Supplementary material for: Solid cancer-directed CAR T cell therapy that attacks both tumor and immunosuppressive cells via targeting PD-L1
Source: Mol Ther Oncol. 2024 Oct 5;32(4):200891. doi: 10.1016/j.omton.2024.200891 (PMC11532918; doi:10.1016/j.omton.2024.200891)
Supplement: Document S1. Figures S1–S9 and Tables S1 and S2 [file mmc1.pdf]

OMTON, Volume *32*

Supplemental information

Solid cancer-directed CAR T cell therapy that attacks both tumor and immunosuppressive cells via targeting PD-L1

Yan Luo, Martha E. Gadd, Yaqing Qie, Andrea Otamendi-Lopez, Jesus E. Sanchez-Garavito, Mieu M. Brooks, Maria J. Ulloa Navas, Tanya Hundal, Shuhua Li, Vanessa K. Jones, Yanyan Lou, Tushar Patel, Roxana Dronca, Mohamed A. Kharfan-Dabaja, Haidong Dong, Alfredo Quinones-Hinojosa, and Hong Qin

Supplemental Material

Supplemental Tables

Table S1: Characterization of representative batches of MC9999 CAR T-cells derived from healthy donors.

	**Healthy Donor 1**		**Healthy Donor 2**		**Healthy Donor 3**	
	**Non CAR T-cells**	**MC9999 CAR T-cells**	**Non CAR T-cells**	**MC9999 CAR T-cells**	**Non CAR T-cells**	**MC9999 CAR T-cells**
Fold Expansion	52	49	64	58	82	92
Viability(%) ≥70%@Day14	86	78	78	80	81	77
Identity(%) ≥ 80%	99.6	99.5	98.9	99.8	99.4	99.2
Potency(%) ≥ 10%	1.08	28	0.71	40.4	0.54	39.4

Three representative batches of MC9999 CAR T-cells, along with their respective Non-CAR T-cells, were produced using peripheral blood T cells of healthy donors. The product release criteria include fold expansion (≥25), cell viability (≥70%), identity (≥80%, as determined by flow cytometry for CD3 positive cells) and potency (≥10%, as determined by flow cytometry for EGFR positive T cells). The corresponding Non-CAR T-cells served as controls.

Table S2: General clinical information about the GBM patients.

Laboratory-generated Patient ID	Patient age range	Diagnosis, disease stage	IDH-1/2	MDMT methyl-ation	Prior steroid use	Date of surgery	Past medical history	Onset symptoms	Figures
QNS120	Patient in 50s	Primary Glioblastoma, Grade 4	WT	Yes	No	11/09/2017	Atrial fibrillation status post ablation	Memory loss, fatigue, insomnia	Fig 4 A,B,C,D
QNS712	Patient in 70s	Primary Glioblastoma, Grade 4	WT	No	Yes (Dex)	04/27/2021	Atrial fibrillation status post ablation	Speech deficit, imbalance, confusion	Fig 4 A,B,C,D
QNS960	Patient in 70s	Primary Glioblastoma, Grade 4	WT	No	Yes (Dex)	03/02/2023	Scleroderma, Sjögren, primary biliary cholangitis	Seizures	Fig 7 A, B,C
GBM Pt 1	Patient in 50s	Primary Glioblastoma, Grade 4	WT	Yes	Yes (Dex)	06/28/2023	Diabetes, hypertension, asthma, obstructive sleep apnea, iron deficiency anemia	Headache	Fig 8 A,B,C and Supp Fig S4
GBM Pt 2	Patient in 60s	Primary Glioblastoma, Grade 4	WT	Yes	Yes (Dex)	06/28/2023	Reflux, hyperlipemia, hypertension, gout, kidney stones	Memory problems, word searching issues, right arm numbness and tingling, and right-sided clumsiness	Fig 8 A,B,C and Supp Fig S4
GBM Pt 3	Patient in 60s	Primary Glioblastoma, Grade 4	WT	Yes	No	07/13/2023 07/20/2023*	Hypertension, diabetes, seizures, cataracts, rheumatoid arthritis	Disorientation, occipital headaches, reading and concentration issues	Fig 8 A,B,C and Supp Fig S4

Dex = Dexamethasone; *Underwent repeat surgical resection one week after first resection.

Selected clinical information for the GBM patients who provided tumor tissue. Additional information tracks the patient samples that were used in the specific experiments and the corresponding figures.

Supplemental Figures

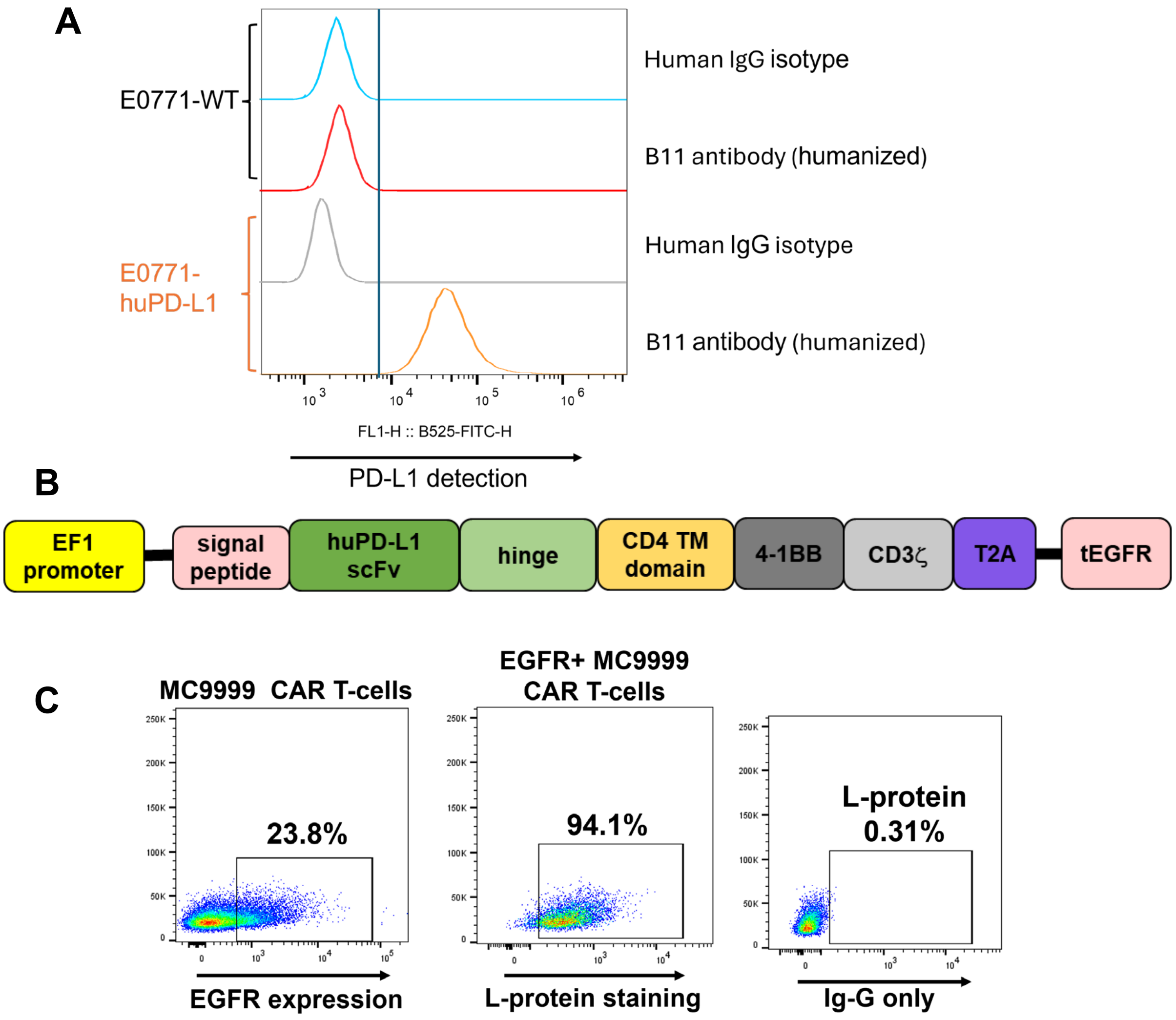

Figure S1. Characterization of MC9999 CAR. (A) Humanized B11 antibody was evaluated against the mouse triple negative breast cancer cell line, E0771.† The humanized anti-PD-L1 antibody did not recognize the mouse PD-L1 expressed by wild-type E0771 but did recognize the E0771 cell line engineered to express human PD-L1. (B) Schematic diagram of MC9999 CAR. The MC9999 CAR is composed of following elements in tandem: EF1 promoter, signal peptide, PD-L1 recognizing element (huPD-L1 scFv), hinge region, transmembrane domain (CD4 TM domain), costimulatory domain (4-1BB), intracellular T cell activation domain (CD3ζ), self-cleaving 2A peptide (T2A), and tEGFR (truncated EGFR that serves as a marker of CAR expression and a suicide switch mediated by cetuximab. (C) The surface expression of the MC9999 CAR in the live EGFR positive T cell population (APC-EGFR, Biosciences) was confirmed using biotinylated L-protein (Genescript, (1μg/ml) per 10^6 cells) followed by PE streptavidin (Biolegend, 5 μl(0.2 mg/ml) per 10^6 cells). IgG only is a negative control.

†Chen C, Li S, Xue J, Qi M, Liu X, Huang Y, Hu J, Dong H, Ling K. PD-L1 tumor-intrinsic signaling and its therapeutic implication in triple-negative breast cancer. JCI Insight. 2021 Apr 22;6(8):e131458. doi: 10.1172/jci.insight.131458. PMID: 33884962

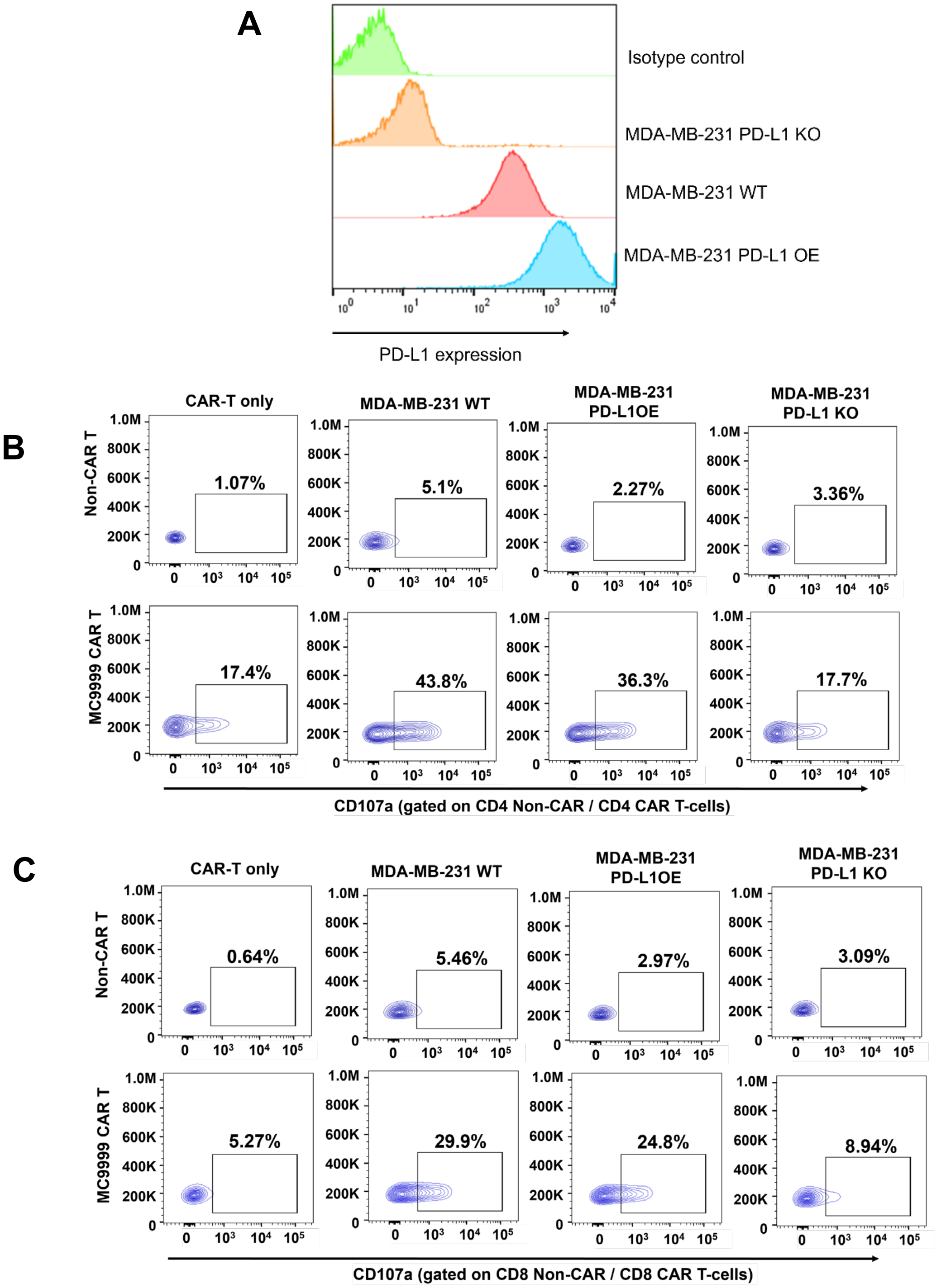

Figure S2. Characterization of PD-L1 expression in MDA-MB-231 cell lines. The wild-type MDA-MB-231 cell line(triple negative breast cancer) was genetically modified to either lose (PD-L1 KO) or overexpress PD-L1 (PD-L1 OE). Validation of PD-L1 expression on these cell lines was conducted using a flow-based immunostaining assay. (A) PD-L1 surface expression of the wild-type MDA-MB-231, MDA-MB-231 PD-L1 KO, and MDA-MB-231 PD-L1 OE was confirmed. In a CD107a degranulation assay, antigen-specific cytotoxicity of CD8 MC9999 CAR T-cells (B) and CD4 MC9999 CAR T-cells (C) was assessed against three MDA-MB-231 cell lines.

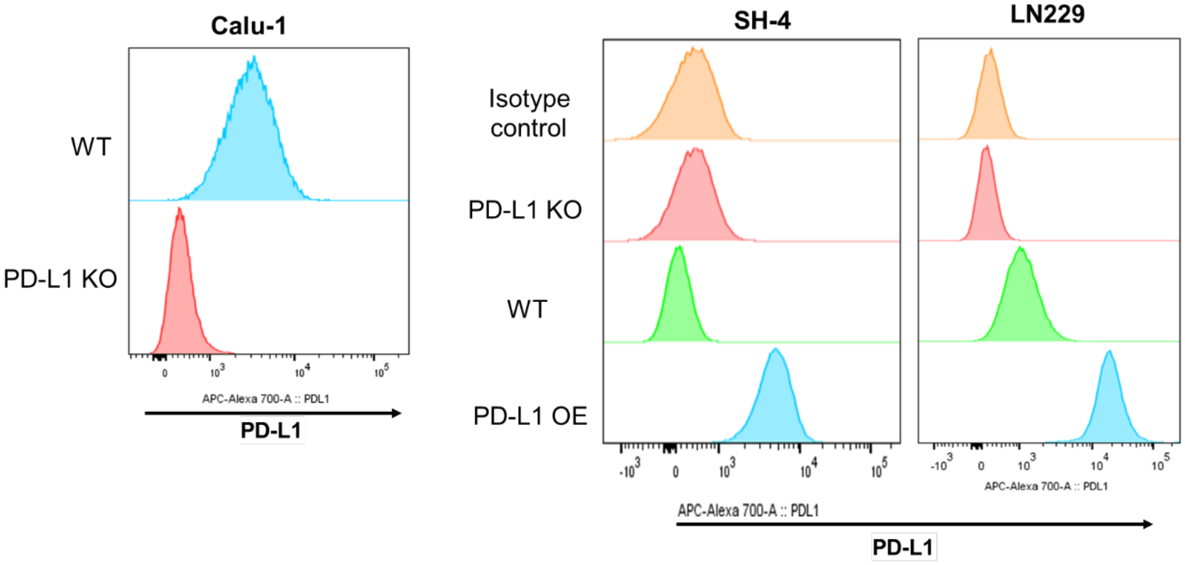

Figure S3. Characterization of PD-L1 expression in various solid tumor cell models. Wild-type Calu-1 (NSCLC), SH-4 (melanoma), and LN229 (GBM) cell lines were genetically modified to either lose (PD-L1 KO) or overexpress PD-L1 (PD-L1 OE). Validation of PD-L1 expression on these cell lines was conducted using a flow-based immunostaining assay.

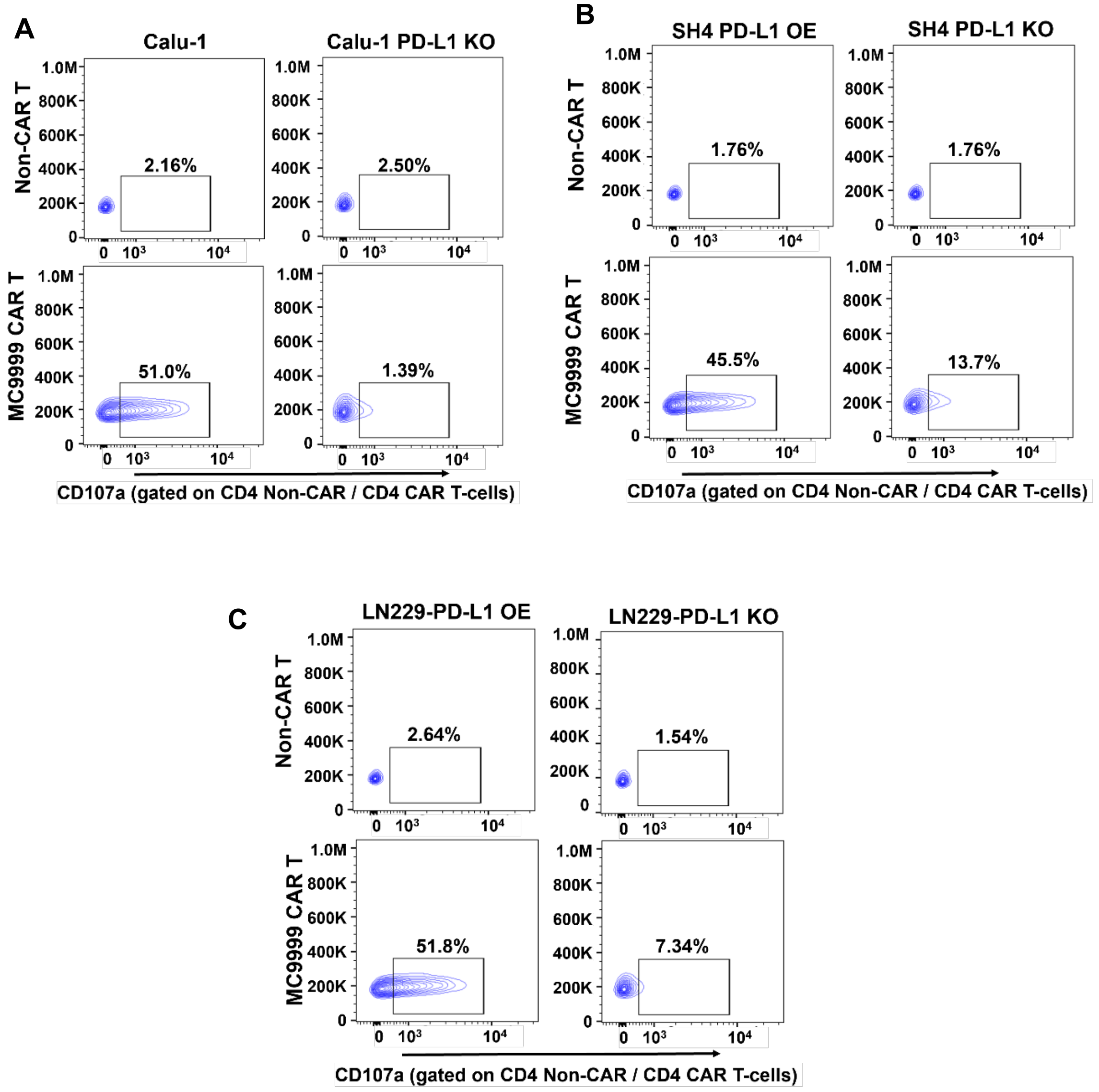

Figure S4. MC9999 CAR T-cells exhibited antigen-specific cytotoxicity against various PD-L1 expressing solid tumors. In a CD107a degranulation assay, antigen-specific cytotoxicity of CD4 MC9999 CAR T-cells was assessed against three solid tumor cell lines: Calu-1 lung cancer (A), SH-4 melanoma (B), and LN229 GBM (C). The corresponding PD-L1-deficient tumor cell variants were included as negative controls.

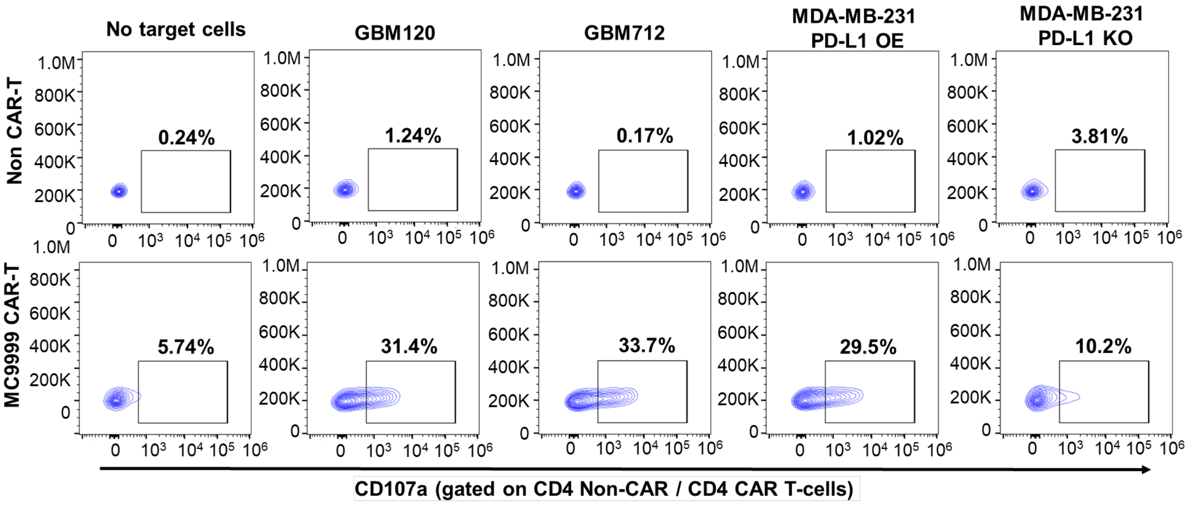

Figure S5. Patient-derived primary GBM cells were targeted by MC9999 CAR T-cells. CD4 MC9999 CAR T-cells were functionally activated by QNS120 and QNS712 tumor cells, as indicated by cell surface staining of CD107a in a degranulation assay. MDA-MB-231 PD-L1 OE and MDA-MB-231 PD-L1 KO cells were used as antigen-positive and antigen-negative controls, respectively.

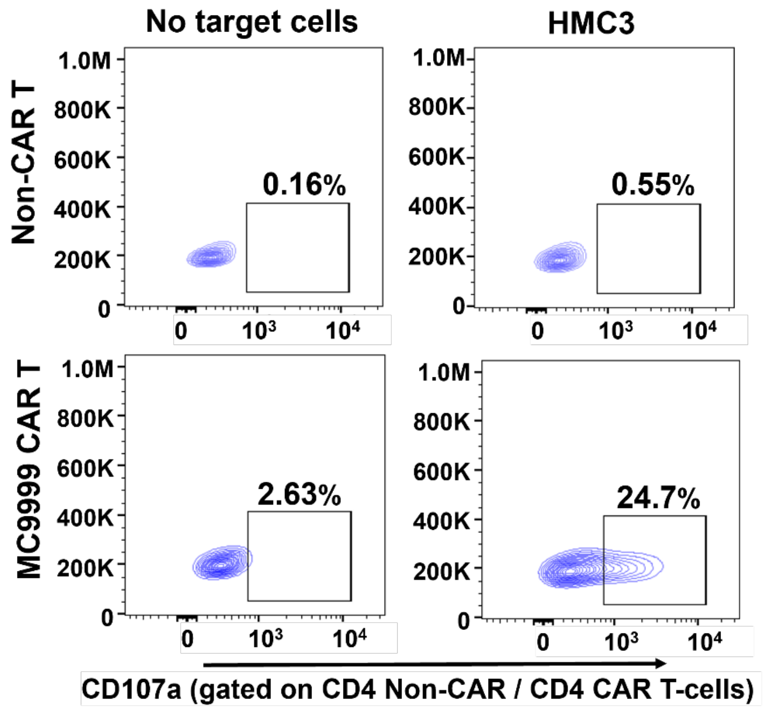

Figure S6. MC9999 CAR T-cells elicited cytolysis on HMC3 cells modeling tumor-associated microglias. Co-incubating MC9999 CAR T-cells with HMC3 cells triggered T-cell degranulation, as evidenced by the cell surface detection of CD107a on the CD4 T cell populations.

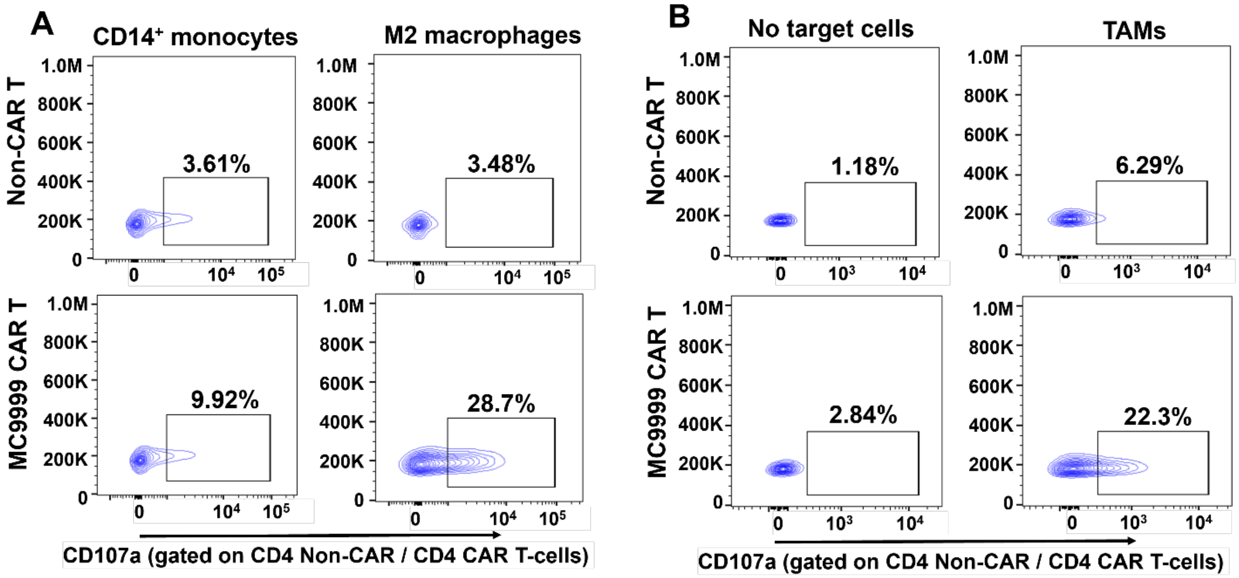

Figure S7. MC9999 CAR T-cells target monocyte-derived M2 macrophages that model immunosuppressive cells as well as patient-derived TAMs. (A) CD4 MC9999 CAR T-cells exhibited cytotoxicity against MDM-M2 macrophages but not CD14$^+$ monocytes, as determined via a CD107a degranulation assay. (B) Evaluation via the CD107a degranulation assay revealed the CD4 MC9999 CAR T-cells, derived from healthy donor T cells, elicited cytotoxicity against the TAMs extracted from GBM tumor.

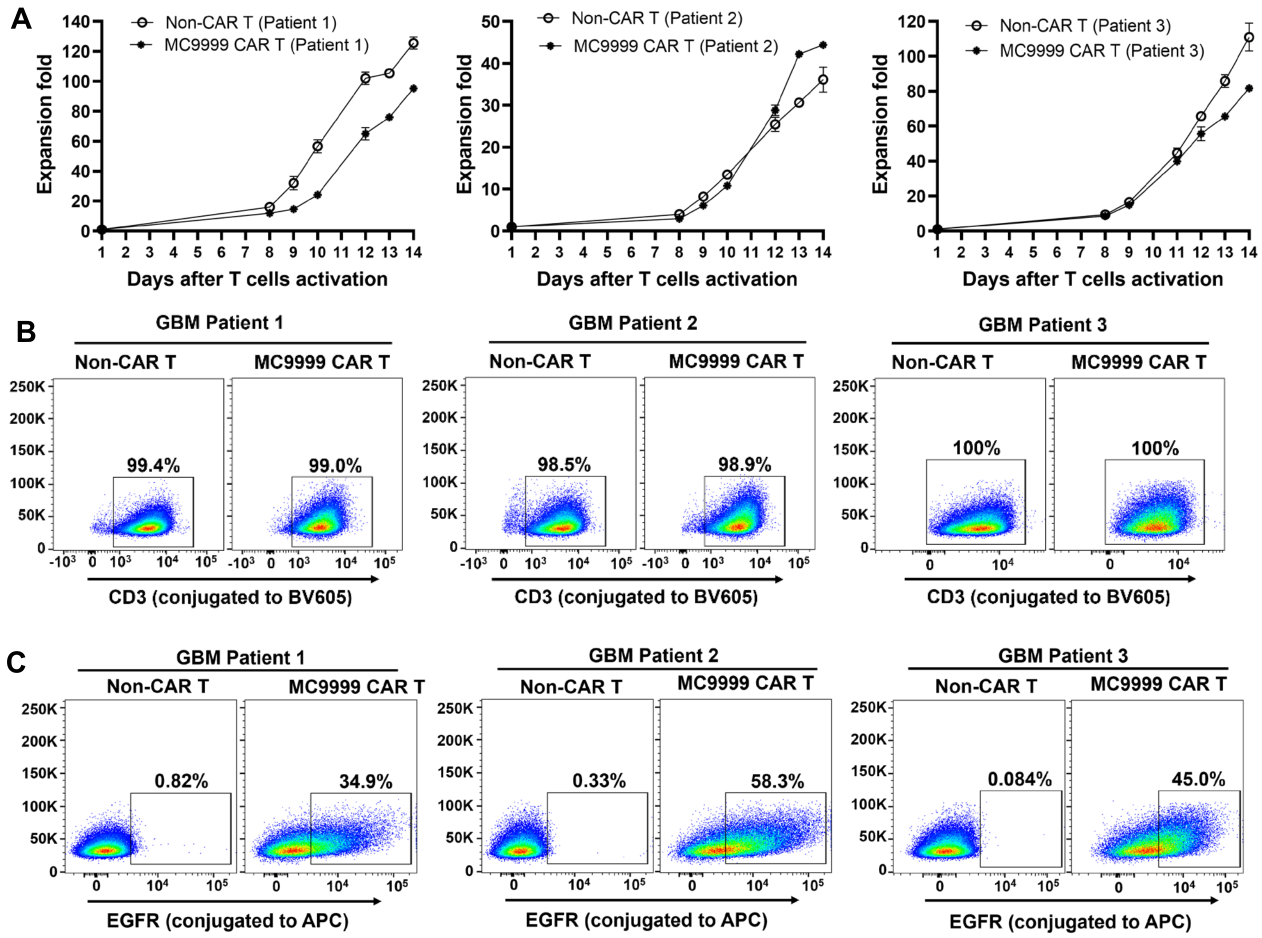

Figure S8. Characterization of the MC9999 CAR T-cells generated from three GBM patients. MC9999 CAR T-cells and their corresponding Non-CAR T-cells were derived from T-cells isolated from peripheral blood of three GBM patients. (A) The growth curves of CAR T-cells over 2-week expansion. Duplicated cell counts were collected for each time point. The produced CAR T-cells were immunostained for CD3 to confirm the identity (B), and EGFR to identify the potency (C).

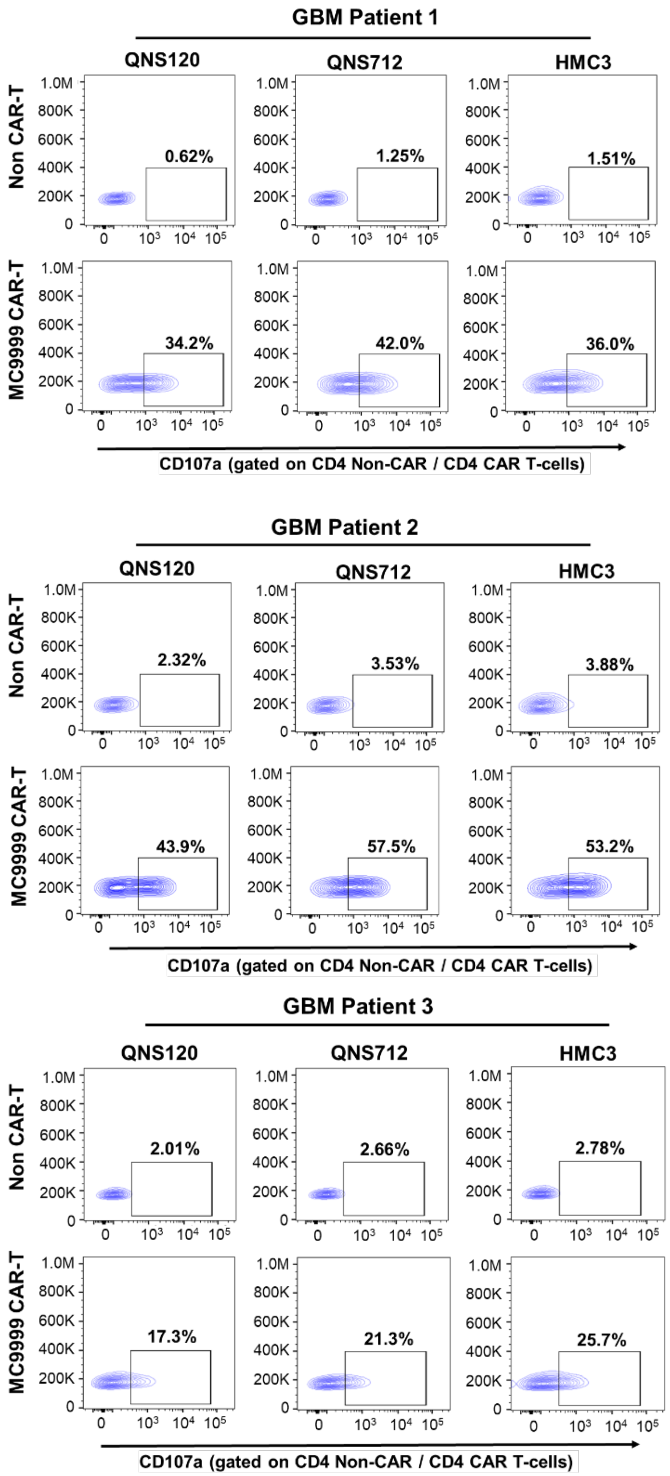

Figure S9. Validation of cytotoxic functionalities of GBM patient-derived MC9999 CAR T-cells. Using peripheral blood T cells obtained from GBM patients, three batches of patient-derived MC9999 CAR T-cells were generated. The cytotoxicity of these patient-derived CD4 CAR T-cells was evaluated through a CD107a degranulation assay. Upon incubation with the PD-L1-expressing target cells including QNS120 and QNS712 GBM patient-derived tumor cells, as well as HMC3 microglia cells, the CD4 CAR T-cells exhibited degranulation activities, as evidenced by the presence of CD107a at the cell surface
